# Multiplexed single-cell profiling of chromatin states at genomic loci by expansion microscopy

**DOI:** 10.1093/nar/gkab423

**Published:** 2021-05-28

**Authors:** Marcus A Woodworth, Kenneth K H Ng, Aaron R Halpern, Nicholas A Pease, Phuc H B Nguyen, Hao Yuan Kueh, Joshua C Vaughan

**Affiliations:** Department of Chemistry, University of Washington, Seattle, WA 98195, USA; Department of Bioengineering, University of Washington, Seattle, WA 98195, USA; Department of Chemistry, University of Washington, Seattle, WA 98195, USA; Department of Bioengineering, University of Washington, Seattle, WA 98195, USA; Department of Bioengineering, University of Washington, Seattle, WA 98195, USA; Department of Bioengineering, University of Washington, Seattle, WA 98195, USA; Department of Chemistry, University of Washington, Seattle, WA 98195, USA; Department of Physiology and Biophysics, University of Washington, Seattle, WA 98195, USA

## Abstract

Proper regulation of genome architecture and activity is essential for the development and function of multicellular organisms. Histone modifications, acting in combination, specify these activity states at individual genomic loci. However, the methods used to study these modifications often require either a large number of cells or are limited to targeting one histone mark at a time. Here, we developed a new method called Single Cell Evaluation of Post-TRanslational Epigenetic Encoding (SCEPTRE) that uses Expansion Microscopy (ExM) to visualize and quantify multiple histone modifications at non-repetitive genomic regions in single cells at a spatial resolution of ∼75 nm. Using SCEPTRE, we distinguished multiple histone modifications at a single housekeeping gene, quantified histone modification levels at multiple developmentally-regulated genes in individual cells, and evaluated the relationship between histone modifications and RNA polymerase II loading at individual loci. We find extensive variability in epigenetic states between individual gene loci hidden from current population-averaged measurements. These findings establish SCEPTRE as a new technique for multiplexed detection of combinatorial chromatin states at single genomic loci in single cells.

## Graphical Abstract

**Graphical Abstract:**
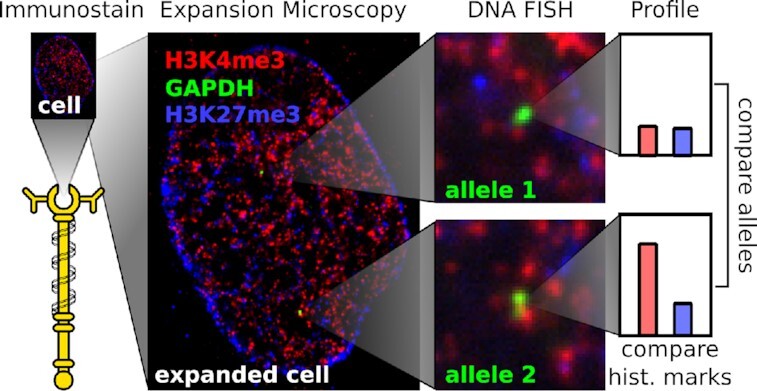
Single cell evaluation of post-translational epigenetic encoding (SCEPTRE) uses expansion microscopy to combine DNA *in situ* labeling with immunofluorescence and quantify histone mark levels at individual loci within cells.

## INTRODUCTION

Proper regulation of genome activity and architecture is critical for development, growth, and function of a multicellular organism (1,2). Regulation occurs in large part at the nucleosome, where ∼147 bp of DNA wrap around an octamer of 4 different histone pairs: H2A, H2B, H3 and H4 (3). Various residues found at the N and C-terminal tails of these histones can acquire post-translational modifications, such as acetylation and methylation, which grant nucleosomes the ability to either participate in organized compaction of chromatin or to recruit transcriptionally relevant protein complexes (4,5). Researchers have therefore suggested that these modifications, also known as histone marks, act as a code for the epigenetic state of genomic regions (6,7). Although several sequencing-based methods are available for studying distinct histone modifications (i.e. ChIP-seq) (8,9), chromatin accessibility (10,11), genomic contact frequencies (12,13), and genomic nuclear locations (14), these methods are either unable to resolve cell-to-cell variations or are limited to studying one histone modification at a time. Therefore, the role these marks play in controlling chromatin structure and gene expression at the single cell and single locus level remains poorly understood and vigorously debated.

To tackle this problem, super-resolution fluorescence microscopy techniques have been used to observe more closely how histone marks impact chromatin organization within a cell's nucleus. Using Stochastic Optical Reconstruction Microscopy (STORM) (15,16), researchers saw that nucleosomes form clusters that vary in size and nuclear distribution depending on a cell's developmental stage or what histone marks they present (17,18). Others have combined STORM with DNA Fluorescence *in situ* hybridization (FISH) to map spatial aspects of genomic loci with a spatial resolution comparable to the observed sizes of these nucleosomal clusters (19). Collectively, these studies suggest that concurrent visualization of DNA and histone modifications with super-resolution microscopy could enable profiling chromatin states at the level of single loci. However, most studies to date have viewed histone marks and genes separately, because combining immunofluorescence and DNA FISH can be challenging due to the harsh solvents and/or high temperatures used in FISH protocols (20–23). Although researchers have visualized immunolabeled histone marks across whole chromosomes (21,22), or at repetitive and highly abundant ALU elements regions labeled with an alternative hybridization strategy (24), there are still no methods available to study multiple histone marks at individual non-repetitive genomic loci at the level of individual nucleosomal clusters. A better understanding of histone mark heterogeneity at individual loci would require a new method capable of further decoupling immunofluorescence and FISH labeling.

We therefore developed a new method, called Single Cell Evaluation of Post-TRanslational Epigenetic Encoding (SCEPTRE), which uses expansion microscopy (ExM) (25,26) to combine DNA FISH with immunofluorescence and quantify histone mark fluorescence signals at individual loci within the nucleus. ExM preserves the signal of antibody labels on protein structures by covalently linking antibodies and proteins to a swellable hydrogel that is grown within the sample (25,26). This signal preservation enables subsequent use of relatively harsh conditions, such as high temperatures and organic solvents, for labeling of genomic DNA by FISH without loss of the antibody signal. At the same time, ExM enables the isotropic expansion of specimens with low distortion so that these specimens may be examined with a high spatial resolution (here ∼75 nm) in the expanded state even when using conventional microscopes with a diffraction-limited resolution of ∼250 nm. We demonstrate the capabilities of SCEPTRE for a variety of systems: (i) we compared signals of multiple histone marks at a housekeeping gene locus; (ii) we distinguished histone mark signals between developmentally-regulated genes in a single cell; (iii) we demonstrate a correlation between histone marks and paused RNA polymerase II in a single region. Together, these experiments establish SCEPTRE as a powerful tool to study the role histone marks have at individual genes within the nuclei of single cells.

## MATERIALS AND METHODS

### Reagents

The following primary antibodies were purchased and used for immunofluorescence: Human anti-centromeres (Antibodies Incorporated, 15-235), Mouse anti-H3K4me3 (EMD Millipore, 05-1339-S), Mouse anti-RNA polymerase II CTD repeat YSPTSPS phosphorylated at Serine 5 (Abcam, ab5408). The following primary antibodies, which were ENCODE-validated (www.encodeproject.org) (27), were purchased and used for immunofluorescence and/or CUT&RUN (28,29) followed by sequencing: Rabbit anti-H3K4me3 (Active motif, 39159), Rabbit anti-H3K27me3 (Active Motif, 39155), Rabbit anti-H3K27ac (Active Motif, 39133), Mouse anti-H3K27me3 (Active Motif, 61017). The following unconjugated secondary antibodies were purchased from Jackson ImmunoResearch: Donkey anti-rabbit (711-005-152) and Donkey anti-human (709-005-149). The following conjugated secondary antibodies were purchased from Jackson ImmunoResearch: Donkey anti-rabbit conjugated with Alexa Fluor 488 (711-545-152) and Donkey anti-mouse conjugated with Alexa Fluor 488 (715-545-150).

The following enzymes were purchased: proteinase K (Thermo Fisher Scientific, EO0491), RNase A (Thermo Fisher Scientific, EN0531), alcohol oxidase (Sigma-Aldrich, A2404-1KU), catalase (Sigma-Aldrich, C100), Phusion Hot-start master mix (New England Biolabs, M0536L), DNase I (New England Biolabs, M0303A) and Maxima H Minus RT Transcriptase (Thermo Fisher Scientific, EP0752).

The following chemical reagents were purchased: 10× phosphate-buffered saline (PBS, Fisher Bioreagents, BP399-1), 32% paraformaldehyde aqueous solution (PFA, Electron Microscopy Sciences, RT15714), 4-(1,1,3,3-tetramethylbutyl)phenyl-polyethylene glycol (Triton X-100, Sigma-Aldrich, X100), Bovine serum albumin (BSA, Rockland Immunochemicals Inc., BSA-50), ATTO 488 NHS-ester (ATTO-TEC GmbH, AD 488-35), Alexa Fluor 568 NHS-ester (Thermo Fisher Scientific, A-20003), methacrylic acid NHS-ester (MA-NHS, Sigma-Aldrich, 730300), 40% acrylamide aqueous solution (Bio-Rad Laboratories, 1610140), 2% bis-acrylamide aqueous solution (Bio-Rad Laboratories, 1610142), 97% sodium acrylate powder (Sigma-Aldrich, 408220), ammonium persulfate (APS, Thermo Fisher Scientific, 17874), tetramethylethylenediamine (TEMED, Thermo Fisher Scientific, 17919), 10× tris-acetate-EDTA (TAE, Fisher Bioreagents, BP2434-4), guanidine hydrochloride powder (Sigma-Aldrich, G3272), sodium azide (Sigma-Aldrich, S2002), poly-L-lysine (Sigma-Aldrich, P8920), sodium bicarbonate (VWR, 470302), formamide (Fisher Chemical, F84-1), 20× saline sodium citrate (SSC, Sigma-Aldrich, S6639), 50% OmniPur Dextran Sulfate (EMD Millipore, 3730), Tween 20 (Sigma-Aldrich, P9416), Hoechst 33258 (Sigma-Aldrich, B2883-25MG), Tris Base (Fisher scientific, BP152-500), methyl viologen dichloride hydrate (Sigma-Aldrich 856177), l-ascorbic acid (Fisher scientific, A61-25), digitonin (EMD Millipore, 300410), glycogen (VWR, 97063-256), sodium chloride (NaCl, Thermo Fisher Scientific, S271500), Ethylenediaminetetraacetic acid disodium salt dihydrate (EDTA, Sigma-Aldrich, E6635), Ethylene glycol-bis(2-aminoethylether)-*N*,*N*,*N*′,*N*′-tetraacetic acid (EGTA, Sigma-Aldrich, E4378) and calcium chloride dihydrate (VWR, 0556).

Alpha-satellite, *GAPDH* set, adapter and conjugated reporter oligonucleotide probe sets were obtained from Integrated DNA Technologies (IDT). A Precise Synthetic Oligo Pool (SC1966-12) containing probes covering the *MYL6*, *HOXC* and *LINC-PINT* regions was obtained from GenScript (for a list of sequences, see supplementary spreadsheet).

### Cell culture

h-TERT RPE1 cells were cultured and grown to ∼80% confluency using Dulbecco's modified eagle medium (Gibco, 11995065) supplemented with 100 units/ml of penicillin and streptomycin (Gibco, 15140122), 1% nonessential amino acids (Gibco, 11140050), and 10% fetal bovine serum (Gibco, 26140079). Cells were then trypsinized with 0.25% trypsin–EDTA (Gibco, 25200056) and seeded at ∼75,000 cells per well on top of round coverslips (no. 1.5, ∼12 mm diameter) placed within 24-well culture plates. After growing overnight (∼18 h), the cells were briefly rinsed with 1× PBS then fixed with either 4–10% PFA in 1× PBS for 10 min at room temperature (∼22°C), or in cold EtOH:MeOH (1:1) for 6 min at –20°C. Fixed cells were washed three times with 1× PBS, then stored in 1× PBS azide (1× PBS with 3 mM sodium azide) at 4°C before use (see Supplementary table S1 for more details).

### Secondary antibody fluorophore conjugation

Conjugation was performed by mixing 40 μl of a secondary antibody solution at a concentration of 1.3 mg/ml with 5 μl of a 1 M sodium bicarbonate solution, then adding 2–5 μg of an NHS ester functionalized fluorophore. The mixture was left to react for 30 min protected from ambient light and the crude reaction mixture was passed through a NAP-5 column (GE Healthcare Life Sciences, 17085301) for collection and purification of the fluorophore-conjugated secondary antibody. Further characterization of the secondary antibody was done by ultraviolet/visible absorption spectroscopy.

### Immunostaining procedure

The immunostain procedure was adapted from previous protocols (17,18), and goes as follows: fixed RPE1 cells were incubated first in permeabilization solution (1× PBS with 0.1% (v/v) Triton X-100) for 10 min, then washed three times with 1× PBS. After permeabilization, cells were incubated in block solution (1× PBS with 10% (w/v) BSA and 3 mM sodium azide) for 1 h at room temperature, followed by incubation in primary solution (2–5 μg/ml of primary antibodies diluted in block) overnight at 4°C. The sample was washed with block three times (10 min each time), then incubated in secondary solution (2–3 μg/ml of secondary fluorophore-conjugated antibodies in block) for 1–2 h at room temperature. The sample was washed once for 10 min with block, then three times with 1× PBS azide. Samples which had been originally fixed in EtOH:MeOH were post-fixed in 4% PFA in 1× PBS for 10 min, then washed three times with 1× PBS azide. Immunostained samples were either immediately gelled or stored in 1× PBS azide at 4°C for up to ∼1 week for later use (see Supplementary table S1 for more details).

### Cell gelation, digestion and expansion

Expansion microscopy was adapted from a previous protocol (26), and goes as follows: immunolabeled cells were treated with freshly prepared 5 mM MA-NHS in 1× PBS for 10 min, then washed three times with 1× PBS. Cells were incubated in monomer solution (1× PBS with 2 M NaCl, 2.5% (w/w) acrylamide, 0.15% (w/w) *N*,*N*’-methylenebisacrylamide and 8.625% (w/w) sodium acrylate) for 10 min before gelation with 0.15–0.2% (w/v) APS and 0.2% TEMED (w/w) at room temperature for at least 30 min in a sealed container backfilled with nitrogen gas. After polymerization, the cell-embedded hydrogel was gently removed from the 12 mm coverslip, then incubated in digestion solution (1× TAE with 0.5% (v/v) Triton X-100, 0.8 M guanidine HCl and 8 units/ml proteinase K) overnight at 37°C. The digested sample was both washed and expanded by placing the sample in deionized water, which was replaced every 15–20 min for at least three times. Hydrogels were stored in 2× SSC at 4°C, typically up to ∼1 month.

### DNA fluorescence *in situ* hybridization

The general DNA FISH procedure for non-repetitive genomic regions (*GAPDH*, *MYL6, HOXC* and *LINC-PINT*) was adapted from previous protocols (30,31), and goes as follows: Briefly, a small (∼3.5 mm × 3 mm × 2 mm) piece of gel from each expanded cell sample was first incubated in hybridization buffer (2× SSC with 50% (v/v) formamide and 0.1% (v/v) Tween 20) for 10 min at room temperature. Samples were incubated in pre-heated hybridization buffer for 30 min at 60°C. A hybridization mixture (2× SSC with 50% formamide (v/v), 10% dextran sulfate (w/v), 0.1% (v/v) Tween 20, 3 mM sodium azide, ∼10-20 nM oligo probe library per kb of targeted genomic region, and 1–1.5× concentration of oligo reporters and adapters to oligo probe library) specific to each sample was preheated to 90°C for 5–10 min and then added to each sample at an approximate 2:1 volume ratio. Samples were denatured at 90–92.5°C for 2.5–10 min and hybridized at 37–42°C overnight. Samples were washed three times, 15 min each time: first with preheated 2× SSCT (2× SSC with 0.1% (v/v) Tween 20) at 60°C, then with preheated 2× SSCT at 37°C, and lastly with 2× SSCT at room temperature. Samples were stored at 4°C in 0.2× SSCT (0.2× SSC with 0.01% (v/v) Tween 20) until needed (within a week). Samples were fully expanded to ∼4× the original size with deionized water at 4°C, replacing the water twice every 10 min (see Supplementary table S1 for more details).

The DNA FISH procedure for the repetitive alpha-satellite region was done as follows: expanded RPE1 cells were incubated for 1 h at room temperature in 1× PBS. The sample was then incubated in 1× PBS supplemented with 100 μg/ml of RNase A for 1 h at 37°C. After RNA digestion, the sample was incubated in 2× SSCT for 30 min at room temperature. The samples were then incubated in hybridization buffer for 30 min at room temperature. The gel was transferred to a hybridization buffer containing 200 nM of alpha-satellite oligonucleotide probe. The sample was denatured for 15 min at 95°C. Gels were washed once in 20× SSC for 15 min at 37°C, then in 2× SSC for 1 h at 37°C. The samples were incubated in 2× SSC with 200 nM alpha-satellite adapter probe and 600 nM of reporter probe A for 30 min at 37°C. The sample was washed with 20× SSC for 20 min at 37°C and lastly with 2× SSC for 20 min at room temperature. After this, the alpha-satellite sample was expanded to ∼3× the original size by incubating the sample in 0.2× SSC, then a second time in 0.2× SSC with 1 μg/ml of Hoechst 33258 (see Supplementary table S1 for more details).

### Sample mounting and imaging

For expanded samples using Alexa Fluor 750 fluorophore-conjugated reporters, samples were incubated in imaging buffer (10 mM Tris buffer (pH 8) with 1 mM Methyl viologen, 1 mM ascorbic acid and 2% (v/v) MeOH) for 10 min. Before imaging, samples were first adhered to a poly-l-lysine–coated rectangular no. 1.5 coverslip, then they were supplemented with ∼30 units/ml alcohol oxidase and 0.2% (w/v) catalase. Samples that did not have Alexa Fluor 750 were adhered to a poly-l-lysine–coated rectangular no. 1.5 coverslip. All samples were imaged with either a Leica SP5 inverted confocal point scanning microscope at the University of Washington Biology Imaging Facility using a Plan Apo CS 63× 1.2 numerical aperture (NA) water-immersion objective, a homebuilt spinning disk confocal microscope using a Nikon CFI60 Plan Apochromat 60× 1.27 NA water-immersion objective, or a conventional wide-field epifluorescence inverted Nikon Ti-S microscope using a Nikon CFI Plan Apo VC 60× 1.2 NA water-immersion objective (see Supplementary table S1 for more details).

### CUT&RUN H3K4me3, H3K27me3 and H3K27ac profiling

CUT&RUN was performed as previously described (29), with the following adaptations: 250 000 trypsinized RPE1 cells were used per antibody condition. Cells were bound to Concanavalin A coated magnetic beads (Bangs Laboratories, BP531), permeabilized with 0.025% (w/v) digitonin, then incubated overnight with 5 μg of either anti-H3K4me3 (Active Motif, 39159), anti-H3K27me3 (Active Motif, 39155) or anti-H3K27ac (Active Motif, 39133) in 500 μl of solution at 4°C. Cells were washed then incubated with protein A-MNase fusion protein (a gift from S. Henikoff, FHCRC) for 15 min at room temperature. After another wash, cells were incubated with 2 mM calcium chloride for 30 min at 0°C to induce MNase cleavage activity. The reaction was stopped by adding 100 μl of 2× STOP buffer (200 mM NaCl, 20 mM EDTA, 4 mM EGTA, 50 μg/ml RNase A, 50 μg/ml glycogen and 2 pg/ml of yeast spike-in DNA) to each sample. Cleaved histone–DNA complexes were isolated by centrifugation and DNA was extracted with a NucleoSpin PCR Clean-up kit (Macherey-Nagel, 740609).

Library preparation for each CUT&RUN antibody condition was done with a KAPA Hyper Prep Kit (VWR, 89125-040) with the PCR amplification settings adjusted to have simultaneous annealing and extension steps at 60°C for 10 seconds. Library products between 200–300 base pairs were selected using Agencourt AMPure XP beads (Beckman Coulter, A63880) then sequenced with an Illumina MiSeq system at the University of Washington Northwest Genomics Center with paired-end 25 bp sequencing read length and TruSeq primer standard for ∼6 million reads per condition.

Paired-end sequencing reads were aligned separately to human (GRCh38/hg38) and yeast genomes using Bowtie2 (32) with the previously suggested specifications for mapping CUT&RUN sequencing data: (29) –local –very-sensitive-local –no-unal –no-mixed –no-discordant -I 10 -X 700. Alignment results were converted to BAM files with SAMtools (33) and then to BED files with BEDTools (34). Reads were sorted and filtered to remove random chromosomes, then, with BEDTools genomecov, histograms were generated for the mapped reads using spiked-in yeast reads and the number of cells for each condition as scaling factors. The results were visualized using the WashU Epigenome Browser (https://epigenomegateway.wustl.edu/) (35).

### Oligonucleotide probe design and amplification

DNA FISH probes were designed using OligoMiner (36), with standard buffer, length and melting temperature conditions, with the exception of the target *MYL6*, which had the following adaptations: base length between 28–42 nucleotides and melting temperature between 38 and 46°C. Orthogonal DNA sequences, which were previously screened for DNA FISH purposes (19,37), were appended to each probe as adapter/reporter hybridizing regions specific to each gene, along with a primer set for amplification. Designed probes were purchased as part of an oligo pool from GenScript, and the probes were amplified using a T7/Reverse-Transcriptase amplification protocol previously published (31), in an RNase-free environment with the following adaptations: After PCR amplification with a Phusion Hot-start master mix and purification with a DNA Clean & Concentrator-5 kit (Zymo Research, D4013), probes were T7 amplified with a HiScribe T7 Quick High Yield RNA Synthesis Kit (New England BioLabs, E2050S) supplemented with 1.3 units/μl RNaseOUT (ThermoFisher Scientific, 10777019) for 16 h at 37°C. DNA was digested with DNase I for 1 h at 37°C. RNA was purified from the sample by first adding LiCl solution from the HiScribe Kit at a 1:7 ratio to the RNA solution, incubating the solution at −20°C for 30 min and pelleting the precipitated RNA by centrifugation (∼17 000 g) for 15 min at 4°C. The supernatant was removed from the tube and the pellet was washed with 70% EtOH. The RNA pellet was centrifuged (∼17 000 g) for 5 min at 4°C and, after carefully removing the supernatant, the pellet was left to dry for 3 min. The RNA was dissolved in water and ∼50 μg of RNA was added to Maxima H Minus RT buffer with 2.86 units/μl Maxima H Minus RT Transcriptase, 2.3 units/μl RNaseOUT, 1 mM dNTP and 14 μM Forward project primer. The solution was incubated at 50°C for 2 h, then samples were digested with 100 μg/ml RNase A for 1 h at 37°C. After RNase digestion, oligonucleotide probes were purified using a DNA Clean & Concentrator-25 kit (Zymo Research, D4033) with Oligo binding buffer (Zymo Research, D4060-1-10). The final product was assumed to have full yield (for a list of sequences, see supplementary spreadsheet).

### Image processing and analysis for SCEPTRE profiling

Image processing and analysis was performed using MATLAB. First, raw images obtained from immunofluorescence channels were smoothed with a gaussian filter using 1–2 standard deviations within a 3 × 3 × 3 matrix. Smoothed images were contrast adjusted, where background pixel levels were clipped at an adaptively determined threshold for each image set at 2–9 third quartiles away from the median of each image stack histogram. The contrast adjusted images were binarized, either by an Otsu method or a Laplace filter with alpha = 0.2 followed by selection of all negative values. A nuclear mask (generated as described below) was applied to the binarized immunofluorescence channel and, after small components (volume < 20 voxels) were removed, a watershed transformation was applied to the segmented clusters. Features, including mean fluorescence intensity of every immunofluorescence channel and overlap with clusters of other segmented immunofluorescence channels, were identified for each segmented cluster (see Supplementary table S2 for more details). For the first image stack, each step was visually inspected to confirm proper threshold levels.

The nuclear mask was generated by applying the same segmentation process from above to either a Hoechst stain channel or the same immunolabeled channel with a contrast adjustment done with 1–3 third quartiles clipping. The segmented channel was subject to morphological dilation with a sphere of 3 pixel radius, to fuse clusters within the nucleus. The convex hull was computed for the largest component (i.e. the nucleus) after all other clusters were removed. The segmented nucleus was morphologically eroded with the same sphere that was used to morphologically dilate the channel (see Supplementary table S2 for more details). For the first image stack, each step was visually inspected to confirm proper threshold levels.

After segmentation of the nuclear channel and immunofluorescence channels, the FISH raw channel was segmented in the same manner with the following exceptions: (i) the nuclear mask was applied after smoothing and before contrast adjustment; (ii) clipping during contrast adjustment was performed with a threshold of 10–15 third quartiles away from the median; (iii) no watershed transformation was applied to FISH segmented regions; (iv) clusters intersecting the periphery of the nuclear mask (e.g. highly fluorescent contaminant in FISH channel next to nucleus) were removed. Features, including mean fluorescence intensity of every immunofluorescence channel and overlap with each immunofluorescence segmented cluster, were identified for each segmented FISH cluster. Since the segmented FISH channel can contain small and dim clusters that, by visual inspection, do not correspond to the FISH-labeled genomic loci, small clusters (volume < 20–80 voxels) were filtered out before analyses. After segmentation of the FISH channel, randomly selected cubic regions were generated throughout the nuclear region of each image stack with a volume approximately equal to the mean volume of the selected FISH clusters. Mean fluorescence intensities of each immunofluorescence channel were determined for these random clusters (see Supplementary table S2 for more details). For the first image stack, each step was visually inspected to confirm proper threshold levels.

Data obtained from the segmented clusters were inspected using contour and scatter plots with MATLAB built-in functions, or violin plots, using the MATLAB script violinplot (https://github.com/bastibe/Violinplot-Matlab). Contours were smoothed with a gaussian filter using 1 standard deviation within a 5 × 5 matrix. Pearson correlation coefficients were determined using the MATLAB function corrcoef.

### Statistical analyses

Each figure, along with their related supplementary figures, represents an individual experiment where all cells were labeled, expanded, imaged and processed under the same conditions. Cell numbers for each experiment were: 1 (Figure 2), 50 (Figure 3, Supplementary Figure S6 and S8), 52 (Figure 4A, Supplementary Figures S9A and S10A), 38 (Figure 4C, Supplementary Figures S9B and S10B), 54 (Figure 5, Supplementary Figure S13−S14), 1 (Supplementary Figure S3), 36 (Supplementary Figure S4A), 10 (Supplementary Figure S4B), 48 (Supplementary Figure S5), 10 (Supplementary Figure S7), 10 (Supplementary Figure S11), 40 (Supplementary Figures S15 and S16), 20 (Supplementary Figures S17 and S18).

Fluorescence signal, defined as the mean fluorescence intensity for a given immunolabeled channel within a cluster of the same experiment, was used as the main measurement for comparing histone mark or paused RNA polymerase II levels within the segmented clusters of immunolabeled, FISH-labeled or randomly selected regions, or between the distribution of fluorescence signals for each set of clusters. Background fluorescence signal is defined as the mean fluorescence intensity for a given immunolabeled channel within the randomly-selected groups of background voxels for H3K4me3 marks in Figure 3, H3K27me3 marks in Figure 4C, or for both immunolabeled channels in Supplementary Figure S7 and S11. The groups of background voxels were equal in size to the median size of segmented clusters for each channel.

An arbitrary ‘on’ threshold (equal to the 5th percentile of the fluorescent signal found within a respective immunolabeled cluster set) is represented in all graphs excluding violin plots, as a qualitative determinant of high or low fluorescence signal within each set of clusters. Correlation coefficients were determined for each comparison between fluorescence signals within a set of clusters. A right-tailed Wilcoxon rank sum test was used to determine if for a given experiment the median fluorescence signal of a cluster set was significantly higher than the median signal in randomly selected regions or a separate set. All numbers corresponding to fraction of overlap and distance are represented as mean ± standard deviation.

To better understand the detection limitations for immunostaining a particular histone mark, cells were concurrently labeled with an antibody of interest and an alternative antibody, both targeting the same mark. The detection efficiency based on colocalization is defined as the percent of voxels from the clusters of the alternative antibody that intersect with the clusters of the antibody of interest. This colocalization is interpreted as a lower bound measurement of detection efficiency due to the challenges of targeting the same epitope with two competing antibodies. A fluorescence-based analysis was used instead to evaluate the detection efficiency of the antibody of interest. In this analysis, we determine the percent of clusters from the alternative antibody distribution that are above the background (i.e. >99.9% of the background fluorescence signal distribution) for the fluorescence signal of the antibody of interest.

## RESULTS

### SCEPTRE uses ExM to co-localize immunolabeled proteins at DNA FISH labeled genomic regions

**Figure 1.**
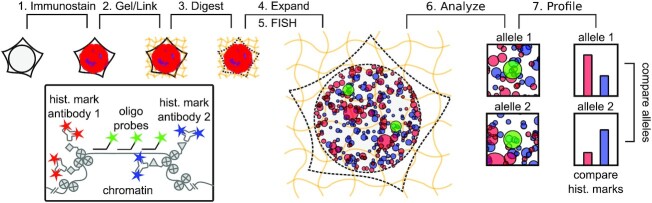
Workflow of SCEPTRE. (**1**) Histone marks or other protein structures are antibody-labeled in fixed cells. (**2**) The sample and antibodies are linked to a swellable hydrogel grown within the sample. (**3**) The sample is digested by proteinase K. (**4**) The hydrogel is expanded in water. (**5**) DNA loci, alleles from the same or different genes, are labeled by FISH. (**6**) The sample is imaged and relevant features are extracted for analysis. **(7)** An epigenetic profile is constructed for each cell, comparing histone mark levels between alleles or genes.

The labeling of individual genomic loci by DNA fluorescence *in situ* hybridization (FISH) has provided a powerful tool for visualizing chromatin structure in single cells (30,38–40). While DNA FISH could be combined with immunofluorescence labeling to enable concurrent visualization of chromatin modification states and associated proteins, integration of these two techniques has been challenging, because the harsh conditions required to melt double-stranded genomic DNA during labeling (e.g., treatment with hot formamide) may remove antibody labels applied before FISH or may compromise the antigenicity of relevant epitopes for post-FISH immunolabeling (Supplementary Figure S1) (20–23). To overcome this challenge, we employed expansion microscopy (ExM) as a means to preserve the signal of immunolabeled protein structures during DNA FISH labeling. In ExM, immunolabeled structures are covalently linked to a swellable hydrogel polymer scaffold that is isotropically expanded in deionized water in order to reveal features closer than the ∼250 nm diffraction limit of light in the expanded state (25,26). ExM not only provides a high spatial resolution (∼75 nm or better when using a standard confocal microscope with ∼4× expanding gels), but also enables antibody labels to be covalently tethered to the hydrogel scaffold, such that DNA FISH can subsequently be performed without loss of antibody fluorescence. ExM has previously been combined with DNA FISH to either visualize the *HER2* gene in tissue (41), or to visualize repetitive centromere regions in plants (42). However, this combination has not yet been used to determine the density of a protein structure, such as histone mark clusters, at specific genomic regions. We refer to this new methodology as Single Cell Evaluation of Post-TRanslational Epigenetic Encoding (SCEPTRE), as a tool to quantify the fluorescence signal of immunolabeled histone marks or proteins structures at individual FISH-labeled genomic loci within individual cells (Figure 1).

**Figure 2.**
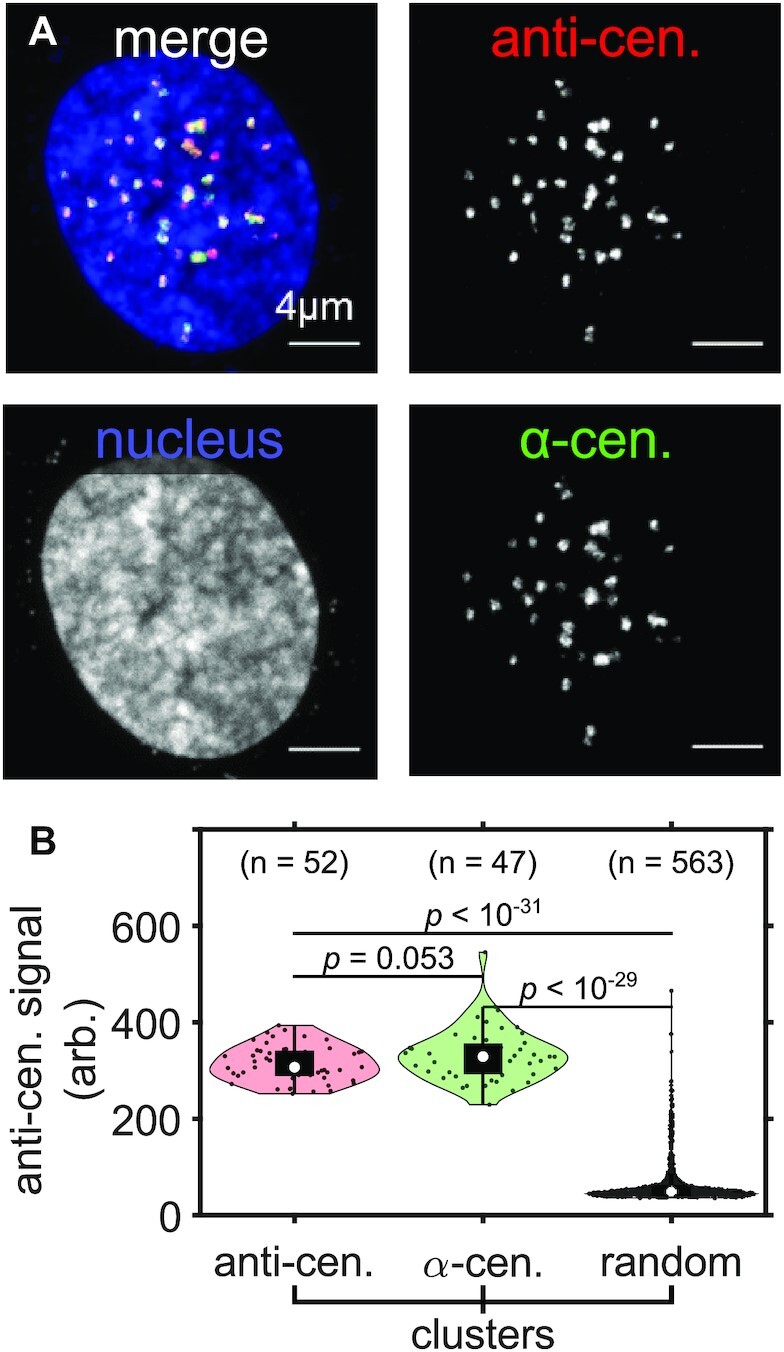
ExM reveals colocalization between centromere-associated proteins with repetitive centromeric DNA. (**A**) Maximum intensity projection image of an entire expanded RPE1 cell nucleus with immunolabeled centromere associated proteins (anti-cen., red), FISH labeled alpha-satellite DNA of centromeres (α-cen., green) and Hoechst-stained nucleus (blue). (**B**) The distribution of anti-centromere fluorescence signal (arb. = arbitrary units) in anti-centromere, α-centromere and in randomly selected region (random) clusters within the nucleus of the cell in (A). Significance determined by a right-tailed Wilcoxon rank-sum test for anti-centromere against random, α-centromere against random, and α-centromere against anti-centromere cluster distributions. All scale bars are in pre-expansion units.

To test the ability of SCEPTRE to report on DNA-protein associations within the nucleus, we immunostained centromere-associated proteins while using DNA FISH to co-stain the repetitive alpha-satellite DNA of centromeres (Figure 2A). ExM images revealed discrete regions corresponding to alpha-centromeres and centromere-associated proteins, as well as significant overlap between these two regions. To quantify this degree of overlap, we created an automated image analysis software routine in MATLAB to segment individual regions corresponding to centromeres and centromere-associated proteins, and then quantified their degree of co-localization (Supplementary Figure S2). From this analysis, we found that DNA-labeled regions had almost complete fractional overlap with centromere-associated proteins (0.97 ± 0.06). Furthermore, the distance between the nearest-neighbor centroids of the protein and DNA labeled regions was small relative to the average radius for either region (77 ± 85 nm versus 234 ± 68 nm (anti-cen.) and 224 ± 65 nm (α-cen.), respectively). We then quantified the fluorescence signal of labeled centromere-associated proteins at individual centromeric DNA clusters, along with randomly selected regions of comparable size to these FISH clusters (Figure 2B). While immunofluorescence and FISH-labeled regions maintained similar anti-centromere fluorescence signals, these regions showed much higher signals compared to randomly selected regions. Therefore, due to the high overlap between the FISH-labeled and immunolabeled regions, the high anti-centromere signal in the FISH-labeled regions, and the fact that the anti-centromere labeled structures did not shift much between pre- and post-expansion (Supplementary Figure S3), we concluded that ExM can co-localize the signal of protein and DNA components of a genomic region within a nucleus.

### SCEPTRE resolves multiple histone modifications at single gene loci in single cells

**Figure 3.**
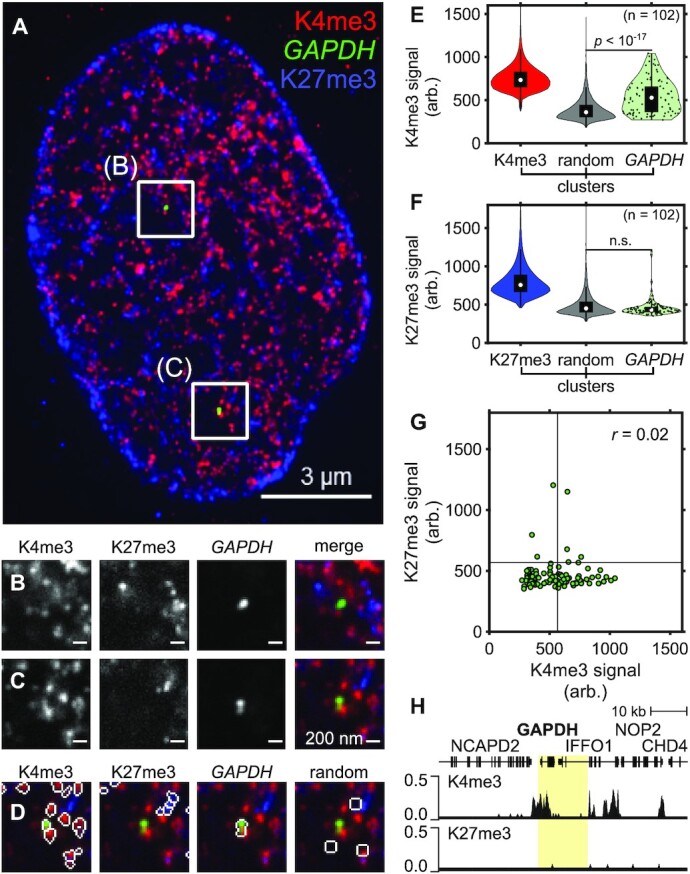
SCEPTRE distinguishes two histone marks at one genomic region. (**A**) An expanded RPE1 cell with immunolabeled H3K4me3 marks (K4me3, red) and H3K27me3 marks (K27me3, blue), and FISH-labeled *GAPDH* (green). (**B**, **C**) Zoomed in views of the approximate center plane of an image stack for each *GAPDH* allele in the cell seen in (A). (**D**) Outline of the segmented regions for H3K4me3, *GAPDH*, H3K27me3 and randomly selected region clusters for the image plane seen in (C). (**E**) Distribution of H3K4me3 fluorescence signal (arb. = arbitrary units) within H3K4me3, randomly selected regions (random) and *GAPDH* clusters. (**F**) Distribution of H3K27me3 fluorescence signal within H3K27me3, randomly selected regions and *GAPDH* clusters. (**G**) H3K27me3 and H3K4me3 fluorescence signals within *GAPDH* clusters (green). Black lines represent the threshold ‘on’ level for each fluorescence signal. The correlation coefficient (*r*) between fluorescence signals within *GAPDH* is shown in the top-right corner of the plot. (**H**) CUT&RUN normalized counts for H3K4me3 (top) and H3K27me3 (bottom) marks in RPE1 cells for the FISH-targeted *GAPDH* region (highlighted). Cluster numbers for (E). and (F). are K4me3 = 343334, K27me3 = 478825, random = 8322, *GAPDH* = 102. Significance determined by a right-tailed Wilcoxon rank-sum test of histone mark fluorescence signals in *GAPDH* against random cluster distributions. All scale bars are in pre-expansion units.

To determine whether SCEPTRE can distinguish between multiple histone marks at a single, non-repetitive genomic region, we concurrently visualized two histone marks, H3K4me3 and H3K27me3, at the house-keeping gene *GAPDH* (Figure 3). *GAPDH* encodes for glyceraldehyde-3-phosphate dehydrogenase, which is highly expressed in many cell types (43) due to its essential role in metabolism; (44) therefore, histone H3K4me3, commonly found at active gene promoters (45), is expected to be present at *GAPDH*, whereas histone H3K27me3, which is associated with repressed regions (46), is expected to be absent. Using SCEPTRE, we measured the fluorescence signals of immunolabeled H3K4me3 and H3K27me3 marks at the FISH-labeled *GAPDH* locus, along with the fraction of overlap between *GAPDH* and H3K4me3 or H3K27me3 clusters (Figure 3).

From this analysis, we observed that *GAPDH* had much higher H3K4me3 fluorescence signal compared to H3K27me3 signal (Figure 3E and F). To our surprise, the H3K4me3 signal found at *GAPDH* varied greatly between loci, with some loci having high signals while others a more baseline level (Figure 3G). These results were the same when only one of the two histone marks was labeled and imaged with *GAPDH* (Supplementary Figure S4), or when a different set of antibodies was used to label H3K4me3 and H3K27me3 in RPE1 cells (Supplementary Figure S5).

Interestingly, histone mark signals were uncorrelated between *GAPDH* alleles in the same cell (Supplementary Figure S6A-B), suggesting histone mark levels at alleles from the same gene are independently regulated. When comparing these fluorescence signals to those obtained from randomly selected regions across the nucleus, *GAPDH* showed lower H3K27me3 signals and much higher H3K4me3 signals than those found at random regions. Similar to the fluorescence signal results, the mean fraction of overlap of *GAPDH* with H3K4me3 clusters was higher than with H3K27me3 clusters (0.21 ± 0.21 versus 0.045 ± 0.11, respectively). To corroborate these results, we measured the density of H3K4me3 and H3K27me3 marks across the RPE1 genome for an ensemble of cells using CUT&RUN followed by sequencing (28,29). Analysis of the CUT&RUN sequencing results revealed that a substantial presence of H3K4me3 marks was found at the targeted *GAPDH* region and only background levels of H3K27me3 marks were found for this same region (Figure 3H), with the closest repressed region observed ∼500 kb away. These results demonstrate that SCEPTRE can distinguish between the abundance of two histone modifications at individual non-repetitive genomic regions within a nucleus.

To determine whether the heterogeneity of H3K4me3 marks at *GAPDH* results from detection limitations of the antibody used above (Rb × H3K4me3), we concurrently labeled H3K4me3 with Rb × H3K4me3 and an alternative antibody targeting the same mark but stained using a different fluorophore (Ms × H3K4me3, Supplementary Figure S7). As an initial estimate of detection efficiency, we calculated the percent of all voxels segmented for the alternative antibody that also segmented for the antibody of interest. This analysis yielded a nominal detection efficiency of 26% for Rb × H3K4me3; however, this value likely under-estimates the true detection efficiency, as the two antibodies likely compete for the same epitope (i.e. the methylated lysine moiety), reducing their ability to co-segment within the same voxel, particularly at high spatial resolution. Therefore, as an alternative measurement of detection efficiency, we directly quantified the cumulative fluorescence signal distribution for Rb × H3K4me3 within clusters segmented with the alternate antibody (Ms × H3K4me3), and we calculated the fraction of this signal above background. This analysis yielded a higher detection efficiency of 88% (Supplementary Figure S7). In contrast, the probability of detecting an antibody signal above background for *GAPDH* loci was considerably lower, at 67% (Supplementary Figure S7E); thus a substantial degree of heterogeneity in H3K4me3 levels at this locus likely reflects biological variability rather than limitations of antibody detection.

H3K4me3 and H3K27me3 are generally thought to mark distinct chromatin states, though they have been reported to colocalize to form ‘bivalent domains’ on genes primed for transcription.(47,48) We therefore investigated the relationship between H3K4me3 and H3K27me3 clusters across the nuclei of the RPE1 cells. As previously observed (18), H3K27me3 clusters preferentially inhabited the periphery of the nucleus, whereas H3K4me3 clusters were more evenly distributed (Figure 3A). There was a low fraction of spatial overlap between H3K4me3 clusters and H3K27me3 clusters (0.079 ± 0.14 H3K4me3 with H3K27me3, 0.12 ± 0.16 H3K27me3 with H3K4me3). The H3K4me3 fluorescence signal in H3K27me3 clusters, as well as the H3K27me3 signal in H3K4me3 clusters, was substantially low, albeit higher than the distribution of random regions (Supplementary Figure S6C-D). We therefore plotted the frequency of H3K4me3 and H3K27me3 signals within each of the other's histone mark's clusters, along with these signals found in randomly selected regions (Supplementary Figure S8). These plots show that H3K4me3 and H3K27me3 form largely non-overlapping clusters, though there exists a small fraction of clusters having high signal from both histone marks. These results suggest that H3K4me3 and H3K27me3 mostly form disjoint clusters, though a very small fraction may colocalize, similar to what has been observed in other differentiated cell types (49).

### SCEPTRE quantifies histone modification levels at multiple genomic loci in single cells

**Figure 4.**
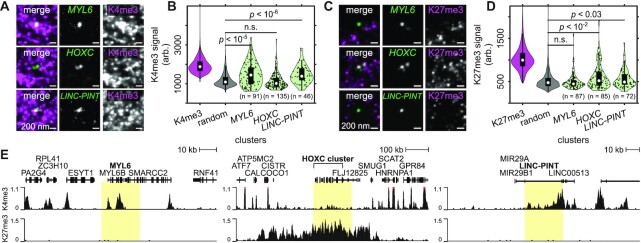
SCEPTRE quantifies one of two histone marks at three genomic regions. (**A**) Example images of the approximate center plane for each image stack of simultaneously FISH-labeled *MYL6*, *HOXC* or *LINC-PINT* loci (green) from the same expanded RPE1 cell immunolabeled for H3K4me3 marks (K4me3, magenta). (**B**) Distribution of H3K4me3 fluorescence signals (arb. = arbitrary units) within H3K4me3, randomly selected regions (random), *MYL6*, *HOXC* and *LINC-PINT* clusters (cluster numbers are K4me3 = 390331, random = 7421, *MYL6* = 91, *HOXC* = 135, *LINC-PINT* = 46). (**C**) Example images of the approximate center plane for each image stack of simultaneously FISH-labeled *MYL6*, *HOXC* or *LINC-PINT* loci (green) from the same expanded RPE1 cell immunolabeled for H3K27me3 marks (K27me3, magenta). (**D**) Distribution of H3K27me3 fluorescence signals within H3K27me3, randomly selected regions, *MYL6*, *HOXC* and *LINC-PINT* clusters (cluster numbers are K27me3 = 196 798, random = 6041, *MYL6* = 87, *HOXC* = 85, *LINC-PINT* = 72). (**E**) CUT&RUN normalized counts for H3K4me3 (top) or H3K27me3 (bottom) at the FISH-labeled *MYL6*, *HOXC* and *LINC-PINT* regions (highlighted). Significance determined by a right-tailed Wilcoxon rank-sum test of fluorescence signals in each FISH-labeled set against the random cluster distribution. All scale bars are in pre-expansion units.

To test whether SCEPTRE can quantify histone mark signals at multiple genomic loci within the same cell, we designed a library of FISH probes to simultaneously label three different genomic regions in RPE1 cells. The first region contains *MYL6*, a gene on Chr12 encoding myosin light chain-6 that is actively expressed in the eye; (50) the second contains the *HOXC* gene cluster, which is normally active in progenitors but repressed upon differentiation; (46,51) the third covers an internal region of long intergenic non-coding P53 induced transcript (*LINC-PINT*) variant, a non-coding transcript that is found on Chr7, which is broadly expressed across multiple tissues (Figure 4) (52). Previous RNA-seq results in RPE1 cells (53) indicated that *MYL6* is expressed at high levels, that all genes within the *HOXC* cluster are silent, and that *LINC-PINT* is transcribed at low levels (Supplementary Figure S3). As expected, bulk analysis of histone modifications at these loci using CUT&RUN revealed H3K4me3 peaks at *MYL6* and *LINC-PINT*, but not within the *HOXC* cluster. H3K27me3 marks, on the other hand, covered a large region encompassing the *HOXC* cluster, but were largely absent from *MYL6* and *LINC-PINT* (Figure 4E).

In agreement with population-level results, H3K4me3 fluorescence signals measured using SCEPTRE were significantly higher at *MYL6* and *LINC-PINT* than at randomly chosen clusters (Figure 4B, *P* < 10^−5^, *MYL6*; *P* <10^−6^, *LINC-PINT*), indicating enrichment of this histone modification at these two loci. Interestingly, both genes showed high H3K4me3 variability between individual loci, similar to what was observed at *GAPDH* loci. In contrast, H3K4me3 signals at the *HOXC* cluster were not significantly higher than those found at random regions. Consistently, H3K4me3 clusters showed greater overlap with the *MYL6* and *LINC-PINT* loci than at the *HOXC* cluster, where it appeared to be visibly excluded (0.23 ± 0.26, *MYL6* and 0.20 ± 0.24, *LINC-PINT* versus 0.068 ± 0.16, *HOXC*). Therefore, SCEPTRE detects differences in H3K4me3 levels between multiple genomic regions seen with population-level analysis, agreeing with the results obtained by CUT&RUN. We note that H3K4me3 mark signals were largely uncorrelated between two alleles of each gene in each cell (Supplementary Figure S9A), as well as between the loci of different genes in the same cell (Supplementary Figure S10A), indicating that the levels of this histone mark are largely independent across loci within individual cells.

Also in agreement with bulk analysis, The *HOXC* cluster showed higher H3K27me3 fluorescence signal compared to random clusters, and higher H3K27me3 signal compared to *MYL6* or *LINC-PINT* (Figure 4D). Strikingly, H3K27me3 levels varied substantially between different *HOXC* clusters, with a substantial fraction of *HOXC* clusters with either low or even baseline levels of H3K27me3, even though all genes in this cluster were silent in their expression (Supplementary Table S3). To determine whether this heterogeneity represents limits in H3K27me3 detection versus *bona fide* biological heterogeneity, we calculated H3K27me3 detection efficiency for this antibody (Rb × H3K27me3, Figure 4C) by co-staining with an alternative antibody, as above (Ms × H3K27me3, Supplementary Figure S11). This analysis yielded a H3K27me3 detection efficiency of 25%, as measured by co-segmentation of the two antibodies, and 72%, determined by quantifying the fraction of Rb × H3K27me3 signal above background for clusters segmented with the alternative antibody (Supplementary Figure S11). In contrast, only 11% of antibody signals at *HOXC* loci were detected above background (Supplementary Figure S11E), suggesting that the majority of H3K27me3 variability at *HOXC* reflects innate biological heterogeneity as opposed to technical limitations of antibody detection.

Although *MYL6* did not have significantly higher H3K27me3 fluorescence levels compared to random regions, *LINC-PINT* did, despite an absence of H3K27me3 marking seen in CUT&RUN data. The presence of H3K27me3 at some *LINC-PINT* loci may reflect the looping of this locus to a different genomic region where H3K27me3 is present; to investigate this possibility, we consulted a previously published Hi-C data set for the RPE1 cell line; (54) indeed *LINC-PINT* maintained high frequency contacts with an adjacent H3K27me3 domain (Supplementary Figure S12), which may contribute to its lower transcriptional levels compared to *MYL6* (Supplementary table S3). These results suggest that, at the current spatial resolution, adjacent genomic regions can influence each other's histone mark levels detected by SCEPTRE. That being said, the SCEPTRE results broadly agree with the results obtained by CUT&RUN and can distinguish between the chromatin modification states of multiple genes in the same cell (e.g. *MYL6* and *HOXC*). As seen with the H3K4me3 marks at these genomic regions, we observed no relationship between the H3K27me3 levels for two alleles of the same gene (Supplementary Figure S9B) or for alleles from different genes (Supplementary Figure S10B) in the same cell.

### H3K4me3 modifications coincide with paused RNA polymerase II at a transcriptionally active locus

**Figure 5.**
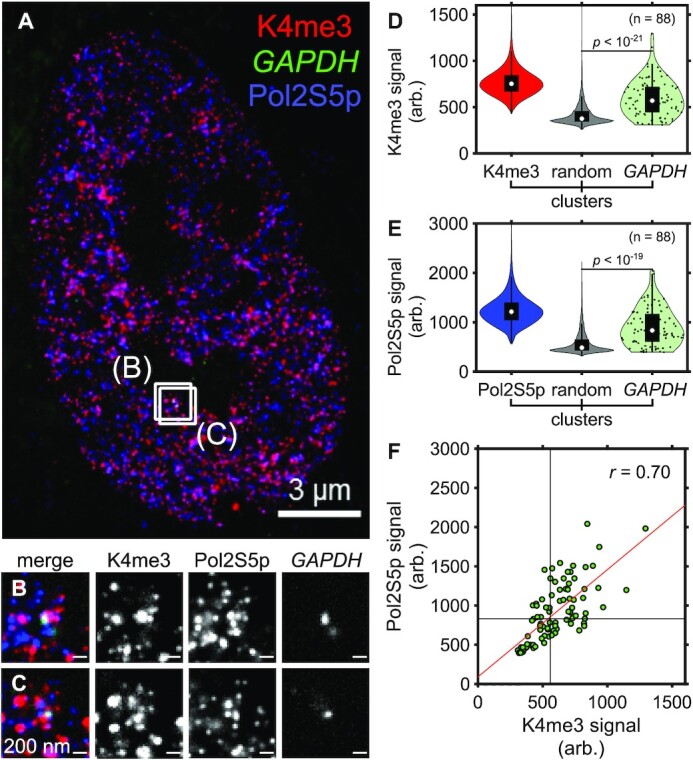
SCEPTRE compares H3K4me3 and paused RNA polymerase II signals at a single genomic region. (**A**) An expanded RPE1 cell with immunolabeled H3K4me3 marks (K4me3, red) and paused RNA polymerase II (Pol2S5p, blue), and FISH-labeled *GAPDH* (green). (**B**, **C**) Zoomed in views of the approximate center plane of an image stack for each *GAPDH* allele in the cell seen in (A). (**D**) Distributions of H3K4me3 fluorescence signal (arb. = arbitrary units) within H3K4me3, randomly selected regions (random) and *GAPDH* clusters. (**E**) Distribution of paused RNA polymerase II fluorescence signal within paused RNA polymerase II, randomly selected regions and *GAPDH* clusters. (**F**) H3K4me3 and paused RNA polymerase II fluorescence signals within *GAPDH* clusters (green). Black lines represent the threshold ‘on’ level for each fluorescence signal, while the red line represents the linear regression. The correlation coefficient (*r*) between fluorescence signals within GAPDH is shown in the top-right corner of the plot. Cluster numbers for (D). and (E). are K4me3 = 440298, Pol2S5p = 542245, random = 8240, *GAPDH* = 88. Significance determined by a right-tailed Wilcoxon rank-sum test of histone mark fluorescence signals in *GAPDH* against random cluster distributions. All scale bars are in pre-expansion units.

H3K4me3 levels have been reported to correlate with active transcription (27) and a model has been proposed where H3K4me3 facilitates the loading of RNA polymerase II, which remains paused proximally to the gene's promoter until a subsequent release step (55). However, this model was based on separate population-level measurements of H3K4me3 and RNA polymerase II, and did not distinguish whether both components coincide directly at the same time at single loci in cells. To test whether both H3K4me3 and paused RNA polymerase II were present simultaneously at *GAPDH*, we performed SCEPTRE with H3K4me3 and the post-translationally modified form of paused RNA polymerase II during transcription initiation, where the Serine 5 of the repeat C-terminal domain of RNA polymerase II is phosphorylated (Figure 5) (56–58).

We detected a large coincidence between H3K4me3 and paused RNA polymerase II, both at the *GAPDH* locus and also more broadly in the nucleus. At individual *GAPDH* loci, there were high signals from both H3K4me3 and paused RNA polymerase II (Figure 5B-E), such that there was also a strong correlation between these signals (Figure 5F, *r* = 0.70). Consistently, *GAPDH* overlapped with both H3K4me3 and paused RNA polymerase II clusters (0.23 ± 0.21 and 0.21 ± 0.19, respectively). Similarly to H3K4me3, paused RNA polymerase II signals were uncorrelated between *GAPDH* loci in the same cell (Supplementary Figure S13). In the nucleus more broadly, there was also substantial colocalization between H3K4me3 clusters and paused RNA polymerase II clusters (Figure 5B, fraction of overlap 0.19 ± 0.21), as well as a strong correlation between these two signals in randomly selected region clusters (Supplementary Figure S14C, *r* = 0.68).

In contrast, no correlation was seen at *GAPDH* between H3K4me3 and H3K27me3 signals (Figure 3G, *r* = 0.02), or between H3K27me3 and paused RNA polymerase II (Supplementary Figure S15F, *r* = 0.04). On a broader level, there was also little to no correlation in random regions between H3K4me3 and H3K27me3 (Supplementary Figure S8C, *r* = 0.18), or between H3K27me3 and paused RNA polymerase II (Supplementary Figure S16C, *r* = 0.17). Interestingly, when H3K27ac, another active histone mark (59), was concurrently visualized with paused RNA polymerase II, some correlation was seen between these two signals, with *r* = 0.43 at *GAPDH* (Supplementary Figure S17F), and *r* = 0.59 at random regions (Supplementary Figure S18C). However, the fraction of *GAPDH* loci with high H3K27ac signals was smaller compared to that with high paused RNA polymerase II signals, suggesting that H3K27ac and the phosphorylation indicative of paused RNA polymerase II play distinct roles in the transcriptional cycle. Together, these results are consistent with a close regulatory relationship between H3K4me3 modifications and the loading of paused RNA polymerase II, both at *GAPDH* and more broadly across the nucleus.

## DISCUSSION

SCEPTRE is a new method capable of profiling chromatin states at multiple genomic loci within the 3D nuclear context of a cell by combining immunofluorescence with DNA *in situ* labeling by means of ExM. This combination enables efficient detection of histone mark fluorescence signals at a resolution of ∼75 nm, sufficient to quantify histone mark abundance at individual genomic loci. In contrast to sequencing-based methods, SCEPTRE provides quantitative measurements of physical properties, such as overlap, density, and position within the nucleus for more than one histone mark at multiple genomic regions. Such measurements reveal a heterogeneity in chromatin states that has been previously masked in ensemble sequencing-based methods.

There are limitations to SCEPTRE compared to other histone mark profiling methods. Sequencing based methods can achieve nucleosome level resolution for histone mark mapping across an entire genome, such as in the case of CUT&RUN (28). Since SCEPTRE relies on DNA FISH, detection of genes by *in situ* labeling often is limited to a minimum labeling size of over 10 kb, since smaller regions are detected with lower efficiency. However, genome organization is thought to occur at a larger scale than that of the single nucleosome. Nucleosomes are known to organize as clusters throughout the nucleus, with spatial sizes ranging around 50–100 nm (17), a size that corresponds to ∼10 kb of genomic DNA, depending on the region's activity state (19). The scale increases further when observing Topologically Associating Domains (TADs), which are genomic regions of ∼200 kb to 1 Mb in size that maintain similar epigenetic and regulatory landscapes (60,61), or the smaller sub-TADs that are ∼185 kb (62), with spatial sizes of ∼160 nm (63). Even larger than 1 Mb are chromatin A and B compartments which are associated with broader open (active) and closed (repressed) states (64), with spatial sizes on the μm scale (65,66). Since SCEPTRE has allowed us to profile multiple genes at the lower scale of this genomic organization, there is potential to build upon this technique in order to target larger genomic regions by using multiplex FISH methods, such as MERFISH (67), seqFISH (68), ORCA (69) or OligoFISSEQ (70). These methods would allow SCEPTRE to interrogate the relationships between histone modifications and gene activity at a variety of developmentally-regulated genes, and at increasingly larger scales of genome organization.

SCEPTRE revealed heterogeneity in the levels of H3K4me3 at active genomic regions such as *GAPDH*, *MYL6* and *LINC-PINT* beyond what would be expected from technical limitations of SCEPTRE. This variability, which has been suggested in sequencing-based single-cell chromatin profiling studies (9,71), suggests that active gene loci can adopt different states with different levels of H3K4me3 modification. Moreover, because SCEPTRE revealed a close correlation between H3K4me3 and paused RNA polymerase II levels at individual loci, this heterogeneity may reflect differences in the transcriptional state of each gene. Given that genes are transcribed in bursts (72), where polymerase recruitment happens intermittently (73), it is plausible that H3K4me3 marks and polymerase II phosphorylation at Serine 5 are concurrently added during a transcriptional burst, but removed at a later stage in the transcriptional cycle. Moving forward, it would be useful to utilize SCEPTRE to further visualize H3K4me3 and other histone marks alongside different stages of transcription, to elucidate how histone marks participate in the regulation of gene transcription.

Similar to H3K4me3, H3K27me3 also appeared to show substantial heterogeneity in its levels at individual *HOXC* loci, with a considerable fraction of loci having low or baseline levels of this modification. As the *HOXC* maintains a transcriptionally silent state in these cells (Supplementary table S3), our results suggest that the *HOXC* cluster is able to maintain a silent chromatin state even with low or baseline levels of H3K27me3 modification. In line with these findings, our recent study has pointed to a necessity for H3K27me3-independent mechanisms in the maintenance of compacted, polymerase-inaccessible state at repressed developmental gene loci (74). Gene repression at the Hox gene cluster requires PRC1, a protein complex that mediates chromatin compaction and gene silencing (75). PRC1 binds to H3K27me3, an interaction that explains the co-localization of these two factors in the genome; however, PRC1 can also bind genomic loci independently of H3K27me3, and thus could conceivably maintain a repressed state at the *HOXC* locus in the absence of H3K27me3 (76). To further investigate these ideas, it will be helpful to use SCEPTRE to interrogate polycomb domains at other genomic loci.

Lastly, there are certain factors that influence the way SCEPTRE profiles the epigenetic state of genes. As demonstrated in the example of the *LINC-PINT* region (Figure 4 and Supplementary Figure S9), the 3D context of a region can influence its epigenetic profile. This ‘crosstalk’ from neighboring regions is most-likely due to the fact that genes with different epigenetic states may be found closer than the resolution of ExM provides at a 4× expansion factor (∼75 nm). If so, methods that achieve better resolution, such as iterative ExM (77) or the combination of ExM with structured illumination microscopy (78), would provide a greater distinction between epigenetic states of neighboring genes while using SCEPTRE. Another factor that may play a role in profiling are the genetic elements within a targeted region. The labeled *LINC-PINT* region was an internal sequence of a gene, which showed a different distribution of H3K4me3 signals compared to the *MYL6* region, whose promoter was found at the center of the labeled region. Therefore, considering the 3D-context of chromatin within cells (seen by Hi-C from a previous study) (54) and the epigenetic landscape of a genomic sequence (seen by CUT&RUN in this study) can help with either selecting each targeted region for SCEPTRE, or in determining the epigenetic state for each region within a cell. Further improvements in multiplexing and resolution would allow SCEPTRE to systematically profile chromatin states in the genome, providing new insights into our understanding of genome structure and function.

## DATA AVAILABILITY

CUT&RUN sequencing data for H3K4me3, H3K27me3 and H3K27ac were submitted to the NCBI gene expression omnibus (http://www.ncbi.nlm.nih.gov/geo/) and are available under the accession number GSE160784. These results, which were obtained using ENCODE-validated antibodies, can be viewed using the following UCSC genome browser session: https://genome.ucsc.edu/s/marcwood/SCEPTRE_RPE1_CnR_hg38. For FISH-labeled regions, CUT&RUN results were confirmed by comparison to previously published ChIP-seq results (79,80).

MATLAB scripts for image processing and analysis are available at GitHub (https://github.com/marcwood13/SCEPTRE_pipeline). Additional data related to this paper will be made available by the corresponding authors upon reasonable request.

## NOTE ADDED IN PROOF

A recently published paper combined immunofluorescence and DNA FISH to visualize multiple histone modifications across many genomic regions using sequential hybridization (81), though imaging was performed using conventional light microscopy that limited their analysis to chromosomal scale domains. This sequential hybridization approach developed can potentially be combined with SCEPTRE to allow for finer-scale profiling of chromatin states across a large number of gene loci.

## Supplementary Material

gkab423_Supplemental_Files

## Notes

Present address: Joshua C. Vaughan, University of Washington, Department of Chemistry, Box 351700, Seattle, WA 98195-1700, USA.

Present address: Hao Yuan Kueh, Department of Bioengineering, 3720 15th Ave NE, Seattle, WA 98195, USA.

## References

[1] Bernstein B.E., Meissner A., Lander E.S. The mammalian epigenome. Cell. 2007; 128:669–681. 17320505 10.1016/j.cell.2007.01.033

[2] Rivera C.M., Ren B. Mapping human epigenomes. Cell. 2013; 155:39–55. 24074860 10.1016/j.cell.2013.09.011 PMC3838898

[3] Kornberg R.D., Lorch Y. Twenty-five years of the nucleosome, fundamental particle of the eukaryote chromosome. Cell. 1999; 98:285–294. 10458604 10.1016/s0092-8674(00)81958-3

[4] Kouzarides T. Chromatin modifications and their function. Cell. 2007; 128:693–705. 17320507 10.1016/j.cell.2007.02.005

[5] Bannister A.J., Kouzarides T. Regulation of chromatin by histone modifications. Cell Res. 2011; 21:381–395. 21321607 10.1038/cr.2011.22 PMC3193420

[6] Strahl B.D., Allis C.D. The language of covalent histone modifications. Nature. 2000; 403:41–45. 10638745 10.1038/47412

[7] Prakash K., Fournier D. Evidence for the implication of the histone code in building the genome structure. Biosystems. 2018; 164:49–59. 29158132 10.1016/j.biosystems.2017.11.005

[8] Barski A., Cuddapah S., Cui K., Roh T.-Y., Schones D.E., Wang Z., Wei G., Chepelev I., Zhao K. High-resolution profiling of histone methylations in the human genome. Cell. 2007; 129:823–837. 17512414 10.1016/j.cell.2007.05.009

[9] Rotem A., Ram O., Shoresh N., Sperling R.A., Goren A., Weitz D.A., Bernstein B.E. Single-cell ChIP-seq reveals cell subpopulations defined by chromatin state. Nat. Biotechnol. 2015; 33:1165–1172. 26458175 10.1038/nbt.3383 PMC4636926

[10] Cusanovich D.A., Daza R., Adey A., Pliner H.A., Christiansen L., Gunderson K.L., Steemers F.J., Trapnell C., Shendure J. Multiplex single-cell profiling of chromatin accessibility by combinatorial cellular indexing. Science. 2015; 348:910–914. 25953818 10.1126/science.aab1601 PMC4836442

[11] Buenrostro J.D., Wu B., Litzenburger U.M., Ruff D., Gonzales M.L., Snyder M.P., Chang H.Y., Greenleaf W.J. Single-cell chromatin accessibility reveals principles of regulatory variation. Nature. 2015; 523:486–490. 26083756 10.1038/nature14590 PMC4685948

[12] Nagano T., Lubling Y., Stevens T.J., Schoenfelder S., Yaffe E., Dean W., Laue E.D., Tanay A., Fraser P. Single-cell Hi-C reveals cell-to-cell variability in chromosome structure. Nature. 2013; 502:59–64. 24067610 10.1038/nature12593 PMC3869051

[13] Ramani V., Deng X., Qiu R., Gunderson K.L., Steemers F.J., Disteche C.M., Noble W.S., Duan Z., Shendure J. Massively multiplex single-cell Hi-C. Nat. Methods. 2017; 14:263–266. 28135255 10.1038/nmeth.4155 PMC5330809

[14] Kind J., Pagie L., Ortabozkoyun H., Boyle S., de Vries S.S., Janssen H., Amendola M., Nolen L.D., Bickmore W.A., van Steensel B. Single-cell dynamics of genome-nuclear lamina interactions. Cell. 2013; 153:178–192. 23523135 10.1016/j.cell.2013.02.028

[15] Rust M.J., Bates M., Zhuang X. Sub-diffraction-limit imaging by stochastic optical reconstruction microscopy (STORM). Nat. Methods. 2006; 3:793–796. 16896339 10.1038/nmeth929 PMC2700296

[16] Huang B., Jones S.A., Brandenburg B., Zhuang X. Whole-cell 3D STORM reveals interactions between cellular structures with nanometer-scale resolution. Nat. Methods. 2008; 5:1047–1052. 19029906 10.1038/nmeth.1274 PMC2596623

[17] Ricci M.A., Manzo C., García-Parajo M.F., Lakadamyali M., Cosma M.P. Chromatin fibers are formed by heterogeneous groups of nucleosomes in vivo. Cell. 2015; 160:1145–1158. 25768910 10.1016/j.cell.2015.01.054

[18] Xu J., Ma H., Jin J., Uttam S., Fu R., Huang Y., Liu Y. Super-resolution imaging of higher-order chromatin structures at different epigenomic states in single mammalian cells. Cell Rep. 2018; 24:873–882. 30044984 10.1016/j.celrep.2018.06.085 PMC6154382

[19] Boettiger A.N., Bintu B., Moffitt J.R., Wang S., Beliveau B.J., Fudenberg G., Imakaev M., Mirny L.A., Wu C., Zhuang X. Super-resolution imaging reveals distinct chromatin folding for different epigenetic states. Nature. 2016; 529:418–422. 26760202 10.1038/nature16496 PMC4905822

[20] Mongelard F., Vourc’h C., Robert-Nicoud M., Usson Y. Quantitative assessment of the alteration of chromatin during the course of FISH procedures. Fluorescent in situ hybridization. Cytometry. 1999; 36:96–101. 10554156 10.1002/(sici)1097-0320(19990601)36:2<96::aid-cyto2>3.3.co;2-o

[21] Chaumeil J., Okamoto I., Heard E. X-chromosome inactivation in mouse embryonic stem cells: analysis of histone modifications and transcriptional activity using immunofluorescence and FISH. Methods in Enzymology, Chromatin and Chromatin Remodeling Enzymes, Part B. 2003; 376:Academic Press 405–419. 10.1016/S0076-6879(03)76027-3 14975321

[22] Cremer M., Grasser F., Lanctôt C., Müller S., Neusser M., Zinner R., Solovei I., Cremer T. Hancock R. Multicolor 3D fluorescence in situ hybridization for imaging interphase chromosomes. The Nucleus: Volume 1: Nuclei and Subnuclear Components, Methods in Molecular Biology. 2008; Totowa, NJ Humana Press 205–239. 10.1007/978-1-59745-406-3_15 18951171

[23] Nehmé B., Henry M., Mouginot D. Combined fluorescent in situ hybridization and immunofluorescence: limiting factors and a substitution strategy for slide-mounted tissue sections. J. Neurosci. Methods. 2011; 196:281–288. 21276820 10.1016/j.jneumeth.2011.01.018

[24] Krufczik M., Sievers A., Hausmann A., Lee J.-H., Hildenbrand G., Schaufler W., Hausmann M. Combining low temperature fluorescence DNA-hybridization, Immunostaining, and super-resolution localization microscopy for nano-structure analysis of ALU elements and their influence on chromatin structure. Int. J. Mol. Sci. 2017; 18:1005. 10.3390/ijms18051005 PMC5454918 28481278

[25] Chen F., Tillberg P.W., Boyden E.S. Expansion microscopy. Science. 2015; 347:543–548. 25592419 10.1126/science.1260088 PMC4312537

[26] Chozinski T.J., Halpern A.R., Okawa H., Kim H.-J., Tremel G.J., Wong R.O.L., Vaughan J.C. Expansion microscopy with conventional antibodies and fluorescent proteins. Nat. Methods. 2016; 13:485–488. 27064647 10.1038/nmeth.3833 PMC4929147

[27] Identification and analysis of functional elements in 1% of the human genome by the ENCODE pilot project. Nature. 2007; 447:799–816. 17571346 10.1038/nature05874 PMC2212820

[28] Skene P.J., Henikoff S. An efficient targeted nuclease strategy for high-resolution mapping of DNA binding sites. eLife. 2017; 6:e21856. 28079019 10.7554/eLife.21856 PMC5310842

[29] Skene P.J., Henikoff J.G., Henikoff S. Targeted *in situ* genome-wide profiling with high efficiency for low cell numbers. Nat. Protoc. 2018; 13:1006–1019. 29651053 10.1038/nprot.2018.015

[30] Beliveau B.J., Joyce E.F., Apostolopoulos N., Yilmaz F., Fonseka C.Y., McCole R.B., Chang Y., Li J.B., Senaratne T.N., Williams B.R. et al. Versatile design and synthesis platform for visualizing genomes with Oligopaint FISH probes. Proc. Natl. Acad. Sci. U.S.A. 2012; 109:21301–21306. 23236188 10.1073/pnas.1213818110 PMC3535588

[31] Beliveau B.J., Boettiger A.N., Nir G., Bintu B., Yin P., Zhuang X., Wu C.-ting In situ super-resolution imaging of genomic DNA with OligoSTORM and OligoDNA-PAINT. Super-Resolution Microscopy, Methods in Molecular Biology. 2017; NY Humana Press 231–252. 10.1007/978-1-4939-7265-4_19 PMC5919218 28924672

[32] Langmead B., Salzberg S.L. Fast gapped-read alignment with Bowtie 2. Nat. Methods. 2012; 9:357–359. 22388286 10.1038/nmeth.1923 PMC3322381

[33] Li H., Handsaker B., Wysoker A., Fennell T., Ruan J., Homer N., Marth G., Abecasis G., Durbin R. 1000 Genome Project Data Processing Subgroup The Sequence Alignment/Map format and SAMtools. Bioinforma. Oxf. Engl. 2009; 25:2078–2079. 10.1093/bioinformatics/btp352 PMC2723002 19505943

[34] Quinlan A.R., Hall I.M. BEDTools: a flexible suite of utilities for comparing genomic features. Bioinformatics. 2010; 26:841–842. 20110278 10.1093/bioinformatics/btq033 PMC2832824

[35] Li D., Hsu S., Purushotham D., Sears R.L., Wang T. WashU Epigenome Browser update 2019. Nucleic Acids Res. 2019; 47:W158–W165. 31165883 10.1093/nar/gkz348 PMC6602459

[36] Beliveau B.J., Kishi J.Y., Nir G., Sasaki H.M., Saka S.K., Nguyen S.C., Wu C., Yin P. OligoMiner provides a rapid, flexible environment for the design of genome-scale oligonucleotide in situ hybridization probes. Proc. Natl. Acad. Sci. U.S.A. 2018; 115:E2183–E2192. 29463736 10.1073/pnas.1714530115 PMC5877937

[37] Kishi J.Y., Lapan S.W., Beliveau B.J., West E.R., Zhu A., Sasaki H.M., Saka S.K., Wang Y., Cepko C.L., Yin P. SABER amplifies FISH: enhanced multiplexed imaging of RNA and DNA in cells and tissues. Nat. Methods. 2019; 16:533–544. 31110282 10.1038/s41592-019-0404-0 PMC6544483

[38] Beliveau B.J., Boettiger A.N., Avendaño M.S., Jungmann R., McCole R.B., Joyce E.F., Kim-Kiselak C., Bantignies F., Fonseka C.Y., Erceg J. et al. Single-molecule super-resolution imaging of chromosomes and in situ haplotype visualization using Oligopaint FISH probes. Nat. Commun. 2015; 6:7147. 25962338 10.1038/ncomms8147 PMC4430122

[39] Levsky J.M., Singer R.H. Fluorescence in situ hybridization: past, present and future. J. Cell Sci. 2003; 116:2833–2838. 12808017 10.1242/jcs.00633

[40] Volpi E.V., Bridger J.M. FISH glossary: an overview of the fluorescence in situ hybridization technique. BioTechniques. 2008; 45:385–409. 18855767 10.2144/000112811

[41] Zhao Y., Bucur O., Irshad H., Chen F., Weins A., Stancu A.L., Oh E.-Y., DiStasio M., Torous V., Glass B. et al. Nanoscale imaging of clinical specimens using pathology-optimized expansion microscopy. Nat. Biotechnol. 2017; 35:757–764. 28714966 10.1038/nbt.3892 PMC5548617

[42] Kubalová I., Černohorská M.S., Huranová M., Weisshart K., Houben A., Schubert V. Prospects and limitations of expansion microscopy in chromatin ultrastructure determination. Chromosome Res. 2020; 28:355–368. 32939606 10.1007/s10577-020-09637-y PMC7691311

[43] Barber R.D., Harmer D.W., Coleman R.A., Clark B.J. GAPDH as a housekeeping gene: analysis of GAPDH mRNA expression in a panel of 72 human tissues. Physiol. Genomics. 2005; 21:389–395. 15769908 10.1152/physiolgenomics.00025.2005

[44] Lodish H., Berk A., Zipursky S.L., Matsudaira P., Baltimore D., Darnell J. Oxidation of glucose and fatty acids to CO2. Molecular Cell Biology. 2000; 4th edn.

[45] Bernstein B.E., Kamal M., Lindblad-Toh K., Bekiranov S., Bailey D.K., Huebert D.J., McMahon S., Karlsson E.K., Kulbokas E.J., Gingeras T.R. et al. Genomic maps and comparative analysis of histone modifications in human and mouse. Cell. 2005; 120:169–181. 15680324 10.1016/j.cell.2005.01.001

[46] Ringrose L., Paro R. Epigenetic regulation of cellular memory by the polycomb and trithorax group proteins. Annu. Rev. Genet. 2004; 38:413–443. 15568982 10.1146/annurev.genet.38.072902.091907

[47] Bernstein B.E., Mikkelsen T.S., Xie X., Kamal M., Huebert D.J., Cuff J., Fry B., Meissner A., Wernig M., Plath K. et al. A bivalent chromatin structure marks key developmental genes in embryonic stem cells. Cell. 2006; 125:315–326. 16630819 10.1016/j.cell.2006.02.041

[48] Blanco E., González-Ramírez M., Alcaine-Colet A., Aranda S., Di Croce L. The bivalent genome: characterization, Structure, and regulation. Trends Genet. 2020; 36:118–131. 31818514 10.1016/j.tig.2019.11.004

[49] Kinkley S., Helmuth J., Polansky J.K., Dunkel I., Gasparoni G., Fröhler S., Chen W., Walter J., Hamann A., Chung H.-R. reChIP-seq reveals widespread bivalency of H3K4me3 and H3K27me3 in CD4 + memory T cells. Nat. Commun. 2016; 7:12514. 27530917 10.1038/ncomms12514 PMC4992058

[50] Bora P.S., Bora N.S., Wu X., Kaplan H.J., Lange L.G. Molecular cloning, sequencing, and characterization of smooth muscle myosin alkali light chain from human eye cDNA: homology with myocardial fatty acid ethyl ester synthase-III cDNA. Genomics. 1994; 19:186–188. 8188229 10.1006/geno.1994.1041

[51] Steffen P.A., Ringrose L. What are memories made of? How Polycomb and Trithorax proteins mediate epigenetic memory. Nat. Rev. Mol. Cell Biol. 2014; 15:340–356. 24755934 10.1038/nrm3789

[52] Lai K.-M.V., Gong G., Atanasio A., Rojas J., Quispe J., Posca J., White D., Huang M., Fedorova D., Grant C. et al. Diverse phenotypes and specific transcription patterns in twenty mouse lines with ablated LincRNAs. PLoS One. 2015; 10:e0125522. 25909911 10.1371/journal.pone.0125522 PMC4409293

[53] Harenza J.L., Diamond M.A., Adams R.N., Song M.M., Davidson H.L., Hart L.S., Dent M.H., Fortina P., Reynolds C.P., Maris J.M. Transcriptomic profiling of 39 commonly-used neuroblastoma cell lines. Sci. Data. 2017; 4:170033. 28350380 10.1038/sdata.2017.33 PMC5369315

[54] Darrow E.M., Huntley M.H., Dudchenko O., Stamenova E.K., Durand N.C., Sun Z., Huang S.-C., Sanborn A.L., Machol I., Shamim M. et al. Deletion of DXZ4 on the human inactive X chromosome alters higher-order genome architecture. Proc. Natl. Acad. Sci. 2016; 113:E4504–E4512. 27432957 10.1073/pnas.1609643113 PMC4978254

[55] Gates L.A., Foulds C.E., O’Malley B.W. Histone marks in the ‘drivers seat’: functional roles in steering the transcription cycle. Trends Biochem. Sci. 2017; 42:977–989. 29122461 10.1016/j.tibs.2017.10.004 PMC5701853

[56] Phatnani H.P., Greenleaf A.L. Phosphorylation and functions of the RNA polymerase II CTD. Genes Dev. 2006; 20:2922–2936. 17079683 10.1101/gad.1477006

[57] Sainsbury S., Bernecky C., Cramer P. Structural basis of transcription initiation by RNA polymerase II. Nat. Rev. Mol. Cell Biol. 2015; 16:129–143. 25693126 10.1038/nrm3952

[58] Harlen K.M., Churchman L.S. The code and beyond: transcription regulation by the RNA polymerase II carboxy-terminal domain. Nat. Rev. Mol. Cell Biol. 2017; 18:263–273. 28248323 10.1038/nrm.2017.10

[59] Heintzman N.D., Hon G.C., Hawkins R.D., Kheradpour P., Stark A., Harp L.F., Ye Z., Lee L.K., Stuart R.K., Ching C.W. et al. Histone modifications at human enhancers reflect global cell-type-specific gene expression. Nature. 2009; 459:108–112. 19295514 10.1038/nature07829 PMC2910248

[60] Nora E.P., Lajoie B.R., Schulz E.G., Giorgetti L., Okamoto I., Servant N., Piolot T., van Berkum N.L., Meisig J., Sedat J. et al. Spatial partitioning of the regulatory landscape of the X-inactivation centre. Nature. 2012; 485:381–385. 22495304 10.1038/nature11049 PMC3555144

[61] Dekker J., Heard E. Structural and functional diversity of topologically associating domains. FEBS Lett. 2015; 589:2877–2884. 26348399 10.1016/j.febslet.2015.08.044 PMC4598308

[62] Rao S.S.P., Huntley M.H., Durand N.C., Stamenova E.K., Bochkov I.D., Robinson J.T., Sanborn A.L., Machol I., Omer A.D., Lander E.S. et al. A 3D map of the human genome at kilobase resolution reveals principles of chromatin looping. Cell. 2014; 159:1665–1680. 25497547 10.1016/j.cell.2014.11.021 PMC5635824

[63] Nozaki T., Imai R., Tanbo M., Nagashima R., Tamura S., Tani T., Joti Y., Tomita M., Hibino K., Kanemaki M.T. et al. Dynamic organization of chromatin domains revealed by super-resolution live-cell imaging. Mol. Cell. 2017; 67:282–293. 28712725 10.1016/j.molcel.2017.06.018

[64] Lieberman-Aiden E., van Berkum N.L., Williams L., Imakaev M., Ragoczy T., Telling A., Amit I., Lajoie B.R., Sabo P.J., Dorschner M.O. et al. Comprehensive mapping of long-range interactions reveals folding principles of the human genome. Science. 2009; 326:289–293. 19815776 10.1126/science.1181369 PMC2858594

[65] Wang S., Su J.-H., Beliveau B.J., Bintu B., Moffitt J.R., Wu C., Zhuang X. Spatial organization of chromatin domains and compartments in single chromosomes. Science. 2016; 353:598–602. 27445307 10.1126/science.aaf8084 PMC4991974

[66] Szabo Q., Bantignies F., Cavalli G. Principles of genome folding into topologically associating domains. Sci. Adv. 2019; 5:eaaw1668. 30989119 10.1126/sciadv.aaw1668 PMC6457944

[67] Moffitt J.R., Zhuang X. RNA imaging with multiplexed error-robust fluorescence in situ hybridization (MERFISH). Methods in Enzymology. Elsevier. 2016; 572:1–49. 10.1016/bs.mie.2016.03.020 PMC5023431 27241748

[68] Eng C.-H.L., Lawson M., Zhu Q., Dries R., Koulena N., Takei Y., Yun J., Cronin C., Karp C., Yuan G.-C. et al. Transcriptome-scale super-resolved imaging in tissues by RNA seqFISH+. Nature. 2019; 568:235–239. 30911168 10.1038/s41586-019-1049-y PMC6544023

[69] Mateo L.J., Murphy S.E., Hafner A., Cinquini I.S., Walker C.A., Boettiger A.N. Visualizing DNA folding and RNA in embryos at single-cell resolution. Nature. 2019; 568:49–54. 30886393 10.1038/s41586-019-1035-4 PMC6556380

[70] Nguyen H.Q., Chattoraj S., Castillo D., Nguyen S.C., Nir G., Lioutas A., Hershberg E.A., Martins N.M.C., Reginato P.L., Hannan M. et al. 3D mpping and accelerated super-resolution imaging of the human genome using in situ sequencing. Nat. Methods. 2020; 17:822–832. 32719531 10.1038/s41592-020-0890-0 PMC7537785

[71] Kaya-Okur H.S., Wu S.J., Codomo C.A., Pledger E.S., Bryson T.D., Henikoff J.G., Ahmad K., Henikoff S. CUT&Tag for efficient epigenomic profiling of small samples and single cells. Nat. Commun. 2019; 10:1930. 31036827 10.1038/s41467-019-09982-5 PMC6488672

[72] Raj A., Peskin C.S., Tranchina D., Vargas D.Y., Tyagi S. Stochastic mRNA synthesis in mammalian cells. PLoS Biol. 2006; 4:e309. 17048983 10.1371/journal.pbio.0040309 PMC1563489

[73] Bartman C.R., Hamagami N., Keller C.A., Giardine B., Hardison R.C., Blobel G.A., Raj A. Transcriptional burst initiation and polymerase pause release are key control points of transcriptional regulation. Mol. Cell. 2019; 73:519–532. 30554946 10.1016/j.molcel.2018.11.004 PMC6368450

[74] Pease N.A., Nguyen P.H.B., Woodworth M.A., Ng K.K.H., Irwin B., Vaughan J.C., Kueh H.Y Tunable, division-independent control of gene activation timing by a polycomb switch. Cell Rep. 2021; 34:108888. 33761349 10.1016/j.celrep.2021.108888 PMC8024876

[75] Eskeland R., Leeb M., Grimes G.R., Kress C., Boyle S., Sproul D., Gilbert N., Fan Y., Skoultchi A.I., Wutz A. et al. Ring1B compacts chromatin structure and represses gene expression independent of histone ubiquitination. Mol. Cell. 2010; 38:452–464. 20471950 10.1016/j.molcel.2010.02.032 PMC3132514

[76] Kahn T.G., Dorafshan E., Schultheis D., Zare A., Stenberg P., Reim I., Pirrotta V., Schwartz Y.B. Interdependence of PRC1 and PRC2 for recruitment to Polycomb Response Elements. Nucleic Acids Res. 2016; 44:10132–10149. 27557709 10.1093/nar/gkw701 PMC5137424

[77] Chang J.-B., Chen F., Yoon Y.-G., Jung E.E., Babcock H., Kang J.S., Asano S., Suk H.-J., Pak N., Tillberg P.W. et al. Iterative expansion microscopy. Nat. Methods. 2017; 14:593–599. 28417997 10.1038/nmeth.4261 PMC5560071

[78] Halpern A.R., Alas G.C.M., Chozinski T.J., Paredez A.R., Vaughan J.C. Hybrid structured illumination expansion microscopy reveals microbial cytoskeleton organization. ACS Nano. 2017; 11:12677–12686. 29165993 10.1021/acsnano.7b07200 PMC5752594

[79] Nozawa R.-S., Nagao K., Igami K.-T., Shibata S., Shirai N., Nozaki N., Sado T., Kimura H., Obuse C. Human inactive X chromosome is compacted through a PRC2-independent SMCHD1-HBiX1 pathway. Nat. Struct. Mol. Biol. 2013; 20:566–573. 23542155 10.1038/nsmb.2532

[80] Kang H., Shokhirev M.N., Xu Z., Chandran S., Dixon J.R., Hetzer M.W. Dynamic regulation of histone modifications and long-range chromosomal interactions during postmitotic transcriptional reactivation. Genes Dev. 2020; 34:913–930. 32499403 10.1101/gad.335794.119 PMC7328517

[81] Takei Y., Yun J., Zheng S., Ollikainen N., Pierson N., White J., Shah S., Thomassie J., Suo S., Eng C.-H.L. et al. Integrated spatial genomics reveals global architecture of single nuclei. Nature. 2021; 590:344–350. 33505024 10.1038/s41586-020-03126-2 PMC7878433

