# Supplementary material for: Multiplexed single-cell profiling of chromatin states at genomic loci by expansion microscopy
Source: Nucleic Acids Res. 2021 May 28;49(14):e82. doi: 10.1093/nar/gkab423 (PMC8373070; doi:10.1093/nar/gkab423)
Supplement: gkab423_Supplemental_Files [file gkab423_supplemental_files.zip › SCEPTRE_NAR_supplementary.pdf]

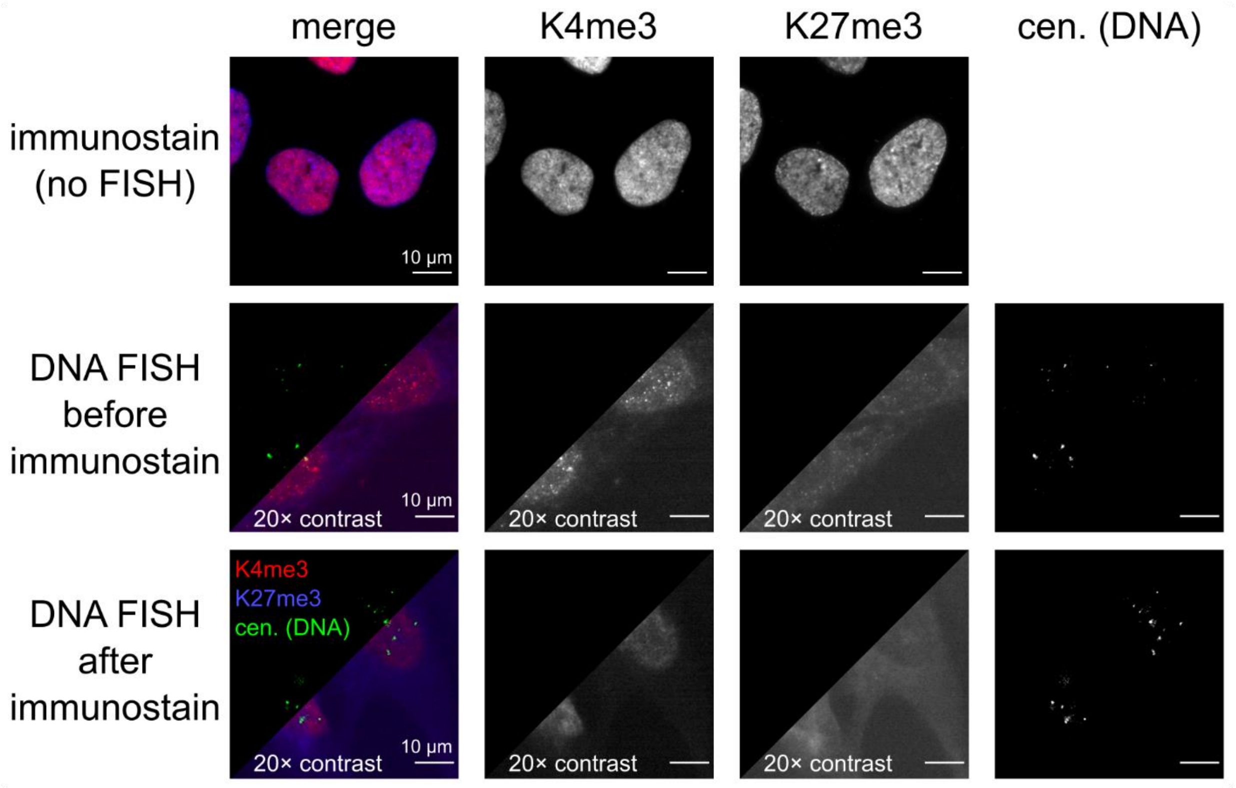

Supplementary figure 1. DNA FISH can disrupt the immunolabeling of nuclear structures. Widefield images of DNA FISH labeled centromeric DNA (green) using hot formamide at 90 °C before (middle row) or after (bottom row) immunolabeling of H3K4me3 (K4me3, red) and H3K27me3 (K27me3, blue). DNA FISH causes a dramatic loss of fluorescent signal for both histone marks compared to immunolabeled cells with no DNA FISH treatment (top row). For the middle and bottom rows, the lower right corner of the histone mark channel images are 20× contrast adjusted compared to the upper left corner of each image and the corresponding top row histone mark image.

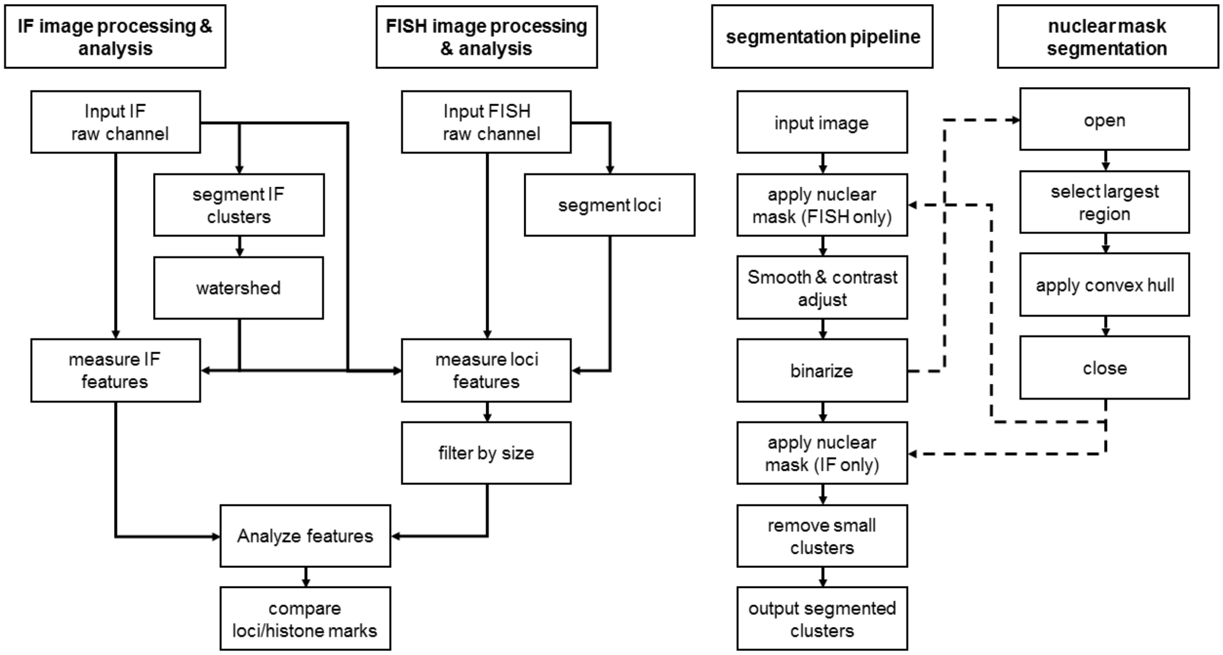

Supplementary figure 2. Image processing schematic for SCEPTRE. Raw images obtained from the immunofluorescence (IF) of protein structures are segmented with the following steps: smooth with a gaussian filter, then contrast adjust with an adaptively determined threshold per cell; binarize either by an Otsu method, or by a Laplace filter followed by selection of all negative values; apply a nuclear mask; apply a watershed transformation. After the segmentation of the nuclear channel and the immunofluorescence channels, the FISH raw channel is then segmented in the same manner with the following exceptions: a nuclear mask is applied after smoothing and before contrast adjustment, and no watershed transformation is applied. Features, including mean fluorescence intensity and fraction of overlap with segmented clusters from each immunofluorescence channel are identified for all segmented clusters within a channel. FISH clusters are further filtered by size. The nuclear mask is generated with the following additional segmentation steps: smooth and contrast adjust either a Hoechst stain channel or one of the present immunofluorescence channels; dilate image to fuse clusters within the nucleus; select largest region encompassing the nucleus; determine the convex hull; erode segmented nucleus; apply to immunofluorescence and FISH channels (for more details, see Materials and Methods and **sup. table 2**).

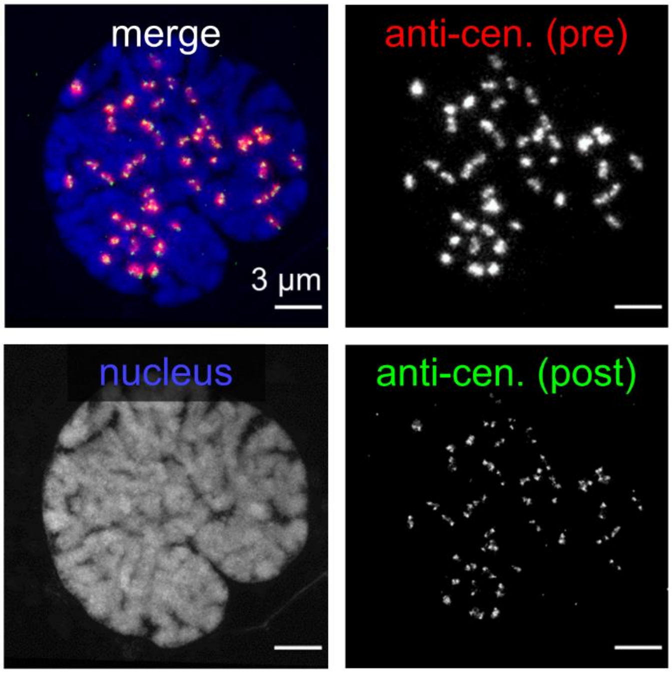

Supplementary figure 3. Correlative imaging of anti-centromere stain before and after expansion. Anti-centromere imaged post-expansion (post, green), is aligned by similarity transform to the same stain imaged pre-expansion (pre, red) and visualized in the context of the post-expansion nucleus labeled by Hoechst (blue). All scale bars are in pre-expansion units*.*

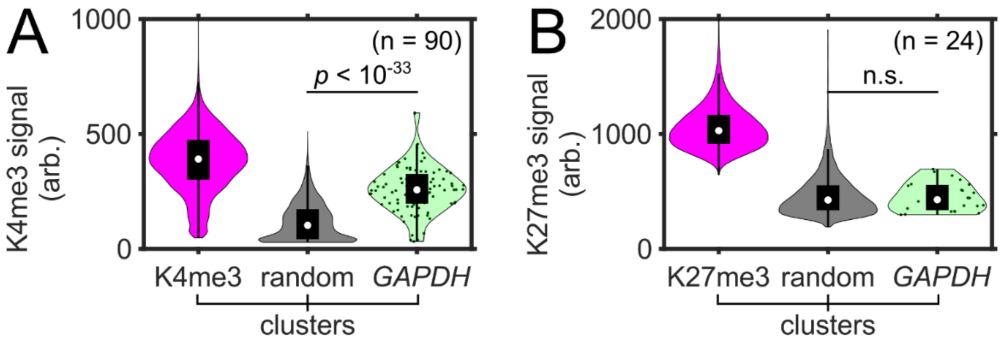

Supplementary figure 4. SCEPTRE measures signal of single-immunolabeled histone marks at *GAPDH* in RPE1 cells. (A) Distributions of H3K4me3 (K4me3) fluorescence signal (arb. = arbitrary units) within H3K4me3, randomly selected regions (random) and *GAPDH* clusters from single-immunolabeled expanded RPE1 cells. Cluster numbers are K4me3 = 196194, random = 5744, *GAPDH* = 90. **(B)** Distribution of H3K27me3 (K27me3) fluorescence signal within H3K27me3, randomly selected regions and *GAPDH* clusters from single-immunolabeled expanded RPE1 cells. Cluster numbers are K27me3 = 60235, random = 6504, *GAPDH* = 24. Significance determined by a right-tailed Wilcoxon rank-sum test of histone mark fluorescence signal in *GAPDH* against random cluster distributions.

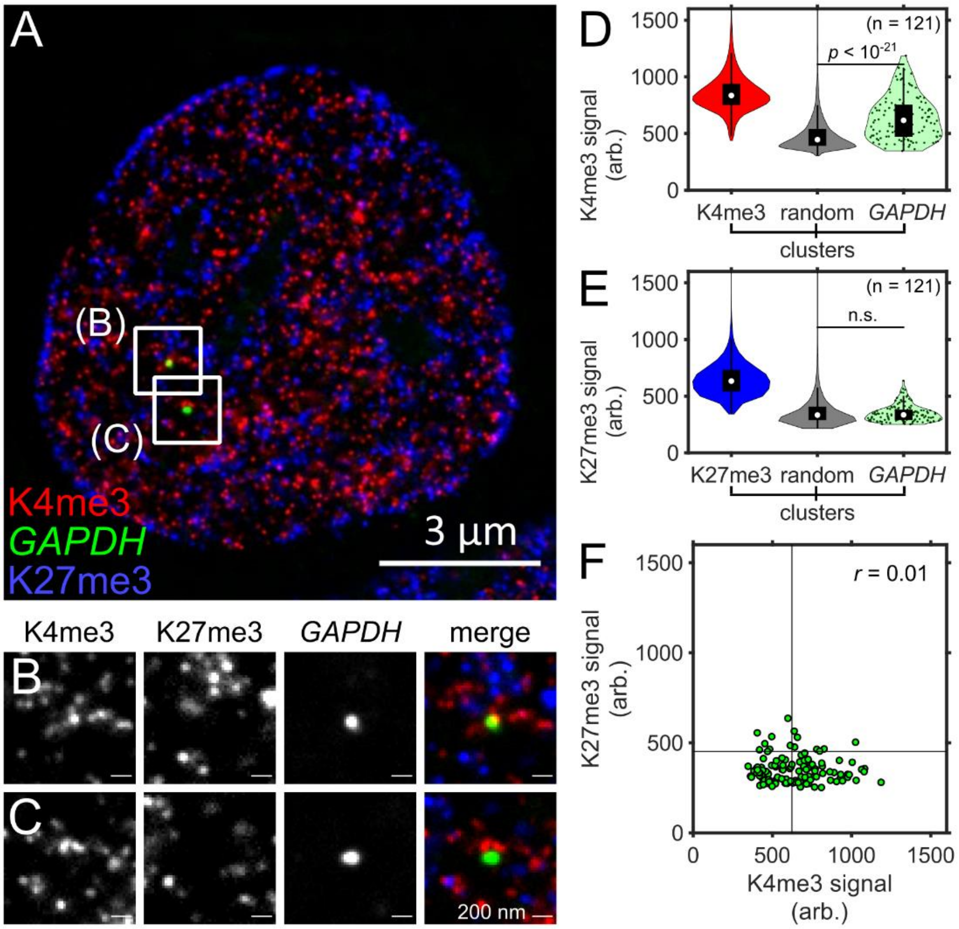

Supplementary Figure 5. SCEPTRE shows reproducible results with a different set of antibodies. (A) An expanded RPE1 cell with immunolabeled H3K4me3 marks (K4me3, red) and H3K27me3 marks (K27me3, blue), and FISH-labeled *GAPDH* (green), using an alternative set of antibodies to **figure 3**. **(B-C)** Zoomed in views of the approximate center plane of an image stack for each *GAPDH* allele in the cell seen in **A. (D)** Distributions of H3K4me3 fluorescence signal (arb. = arbitrary units) within H3K4me3, randomly selected regions (random) and *GAPDH* clusters. **(E)** Distribution of H3K27me3 fluorescence signal within H3K27me3, randomly selected regions and *GAPDH* clusters. **(F)** H3K27me3 and H3K4me3 fluorescence signals within *GAPDH* clusters (green). Black lines represent the threshold "on" level for each fluorescence signal. Cluster numbers for **D.** and **E.** are K4me3 = 250644, K27me3 = 262307, random = 7406, *GAPDH* = 121. The correlation coefficient (*r*) between fluorescence signals within *GAPDH* is shown in the top-right corner of the plot. Significance determined by a right-tailed Wilcoxon rank-sum test of histone mark fluorescence signals in *GAPDH* against random cluster distributions. All scale bars are in pre-expansion units. Although the antibodies used in **figure 3** and for targeting H3K27me3 in **A.** were validated by ENCODE, the H3K4me3 targeting antibody in **A.** was not.

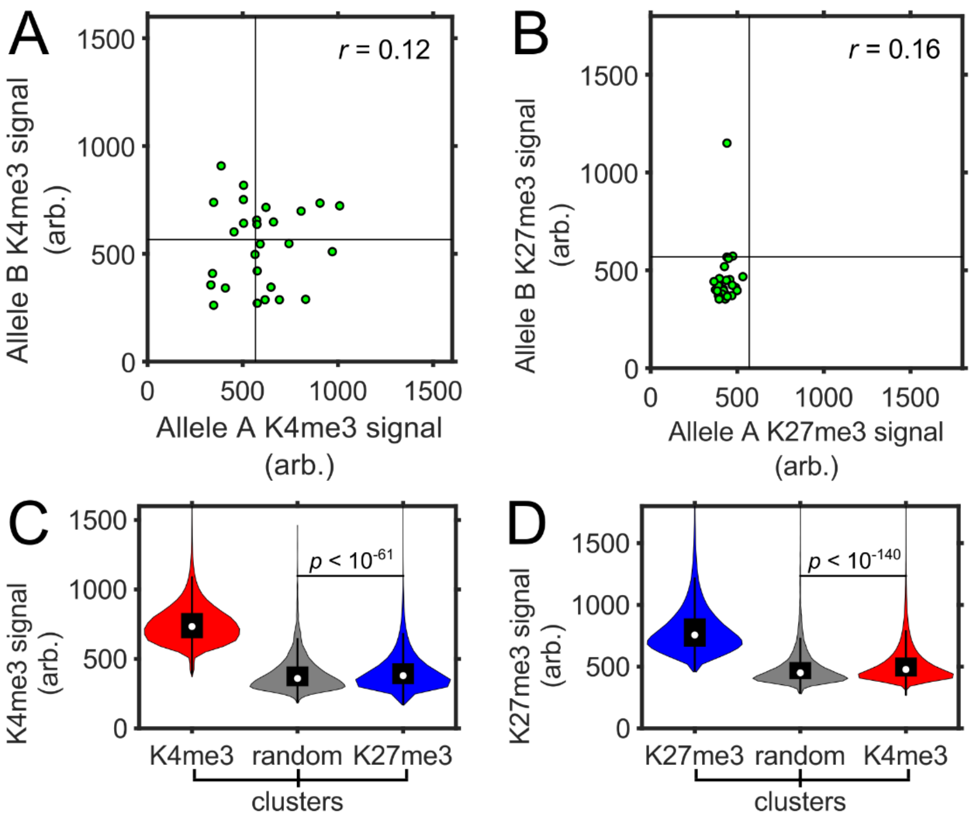

Supplementary figure 6. SCEPTRE compares H3K4me3 and H3K27me3 signals between different *GAPDH* alleles in the same cell, or between histone mark cluster distributions. (A-B) Fluorescence signal (arb. = arbitrary units) of either H3K4me3 (K4me3) in **A.**, or H3K27me3 (K27me3) in **B.**, in *GAPDH* alleles within the same cell from the data set in **figure 3** (one locus from each cell containing 2-4 loci is randomly assigned as allele A, and a second locus as allele B). Black lines represent the threshold "on" level for each histone mark fluorescence signal. The correlation coefficient (*r*) is shown on the top-right corner of each plot. **(C-D)** Fluorescence signal of either H3K4me3 in **C.**, or H3K27me3 in **D.**, for each distribution of H3K4me3 (red), H3K27me3 (blue) and randomly selected region (random, gray) clusters within the cells in **figure 3**. Significance determined by a right-tailed Wilcoxon rank-sum test of fluorescence signals in each histone mark cluster set against random cluster distributions.

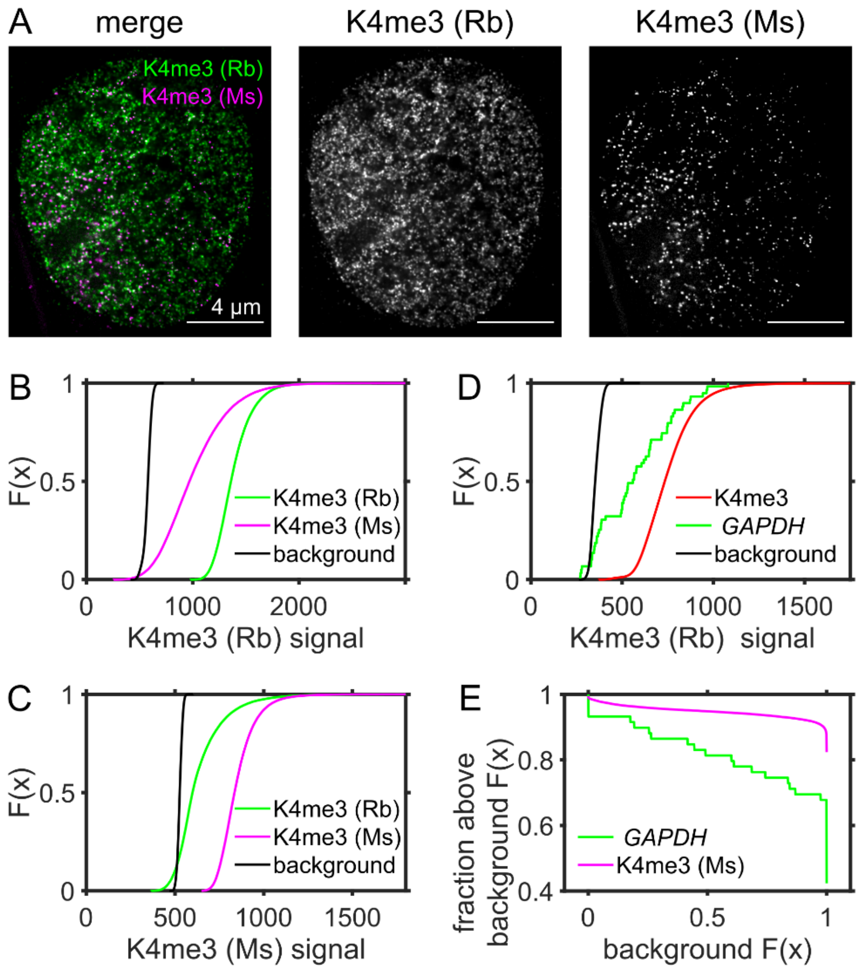

Supplementary figure 7. Dual labeling of H3K4me3 marks with two different antibodies provides information on the detection efficiency of each antibody. (A) An expanded RPE1 cell with H3K4me3 (K4me3) marks immunolabeled simultaneously by two different antibodies: Rb×H3K4me3 (Rb, green) and Ms×H3K4me3 (Ms, magenta). **(B-C)** Empirical cumulative distribution functions (F(x)) for the fluorescence signal of Rb×H3K4me3 in **B.**, Ms×H3K4me3 in **C.**, for Rb×H3K4me3 clusters (green), Ms×H3K4me3 clusters (magenta), or background (black) from the same group of cells as the one in **A.** **(D)** Empirical cumulative distribution functions (F(x)) for the fluorescence signal of Rb×H3K4me3 in *GAPDH* clusters (green), Rb×H3K4me3 clusters (red), or background (black) from the cells in **figure 3.** **(E)** The fraction of either *GAPDH* clusters (green) from **figure 3,** or the Ms×H3K4me3 clusters (magenta) from the same group of cells as the one in **A.**, that are above the Rb×H3K4me3 fluorescence signal of background F(x).

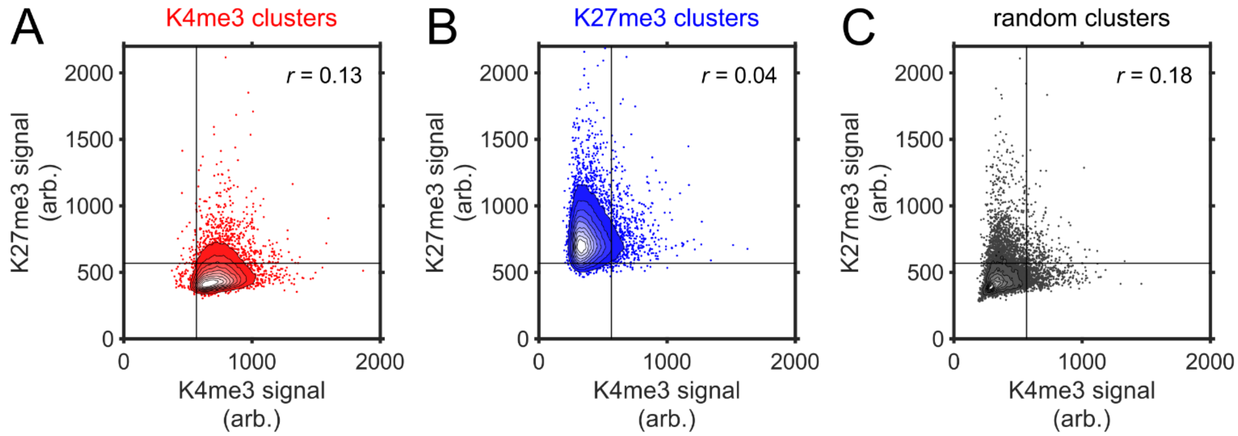

Supplementary figure 8. SCEPTRE compares H3K4me3 and H3K27me3 signals within segmented immunostained and random clusters. (A-C) Contours for the fluorescence signal (arb. = arbitrary units) frequency of H3K4me3 (K4me3) and H3K27me3 (K27me3) in the cluster sets of H3K4me3 (red) in **A.**, H3K27me3 (blue) in **B.**, and randomly selected regions (random, gray) in **C.**. Straight black lines represent the threshold "on" level for each fluorescence signal. Contours have uniformly spaced steps ranging from 0.1 to 0.9 frequency and represent all clusters obtained for cells in **figure 3**. The remaining scatter in **A.** and **B.** is a 100-fold downsample of the original data by random selection for plot representation purposes. Correlation coefficients (*r*) for each data set, which are calculated before downsampling, are shown in the top-right corner of each plot.

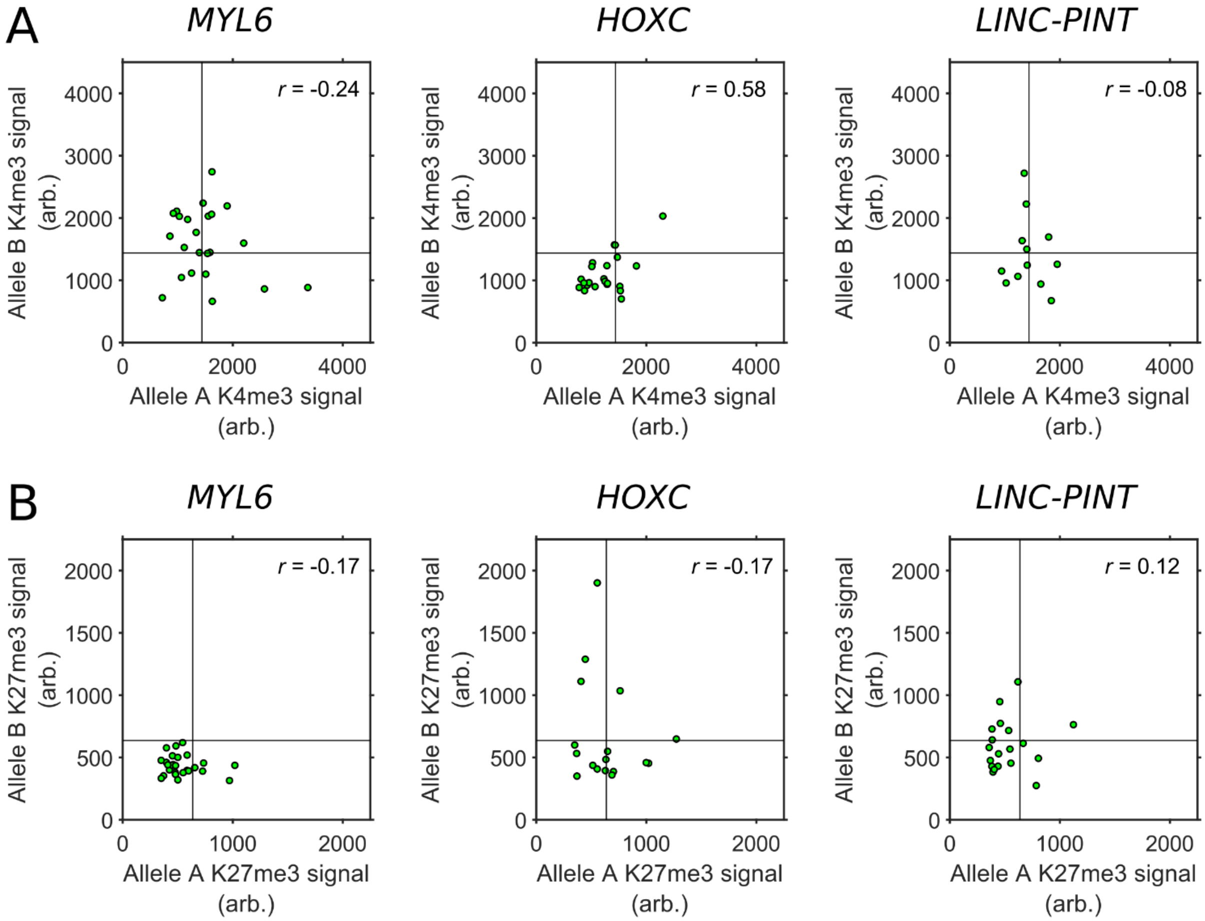

Supplementary figure 9. SCEPTRE compares H3K4me3 or H3K27me3 signals between alleles of one of multiple genes in the same cell. (A-B) Fluorescence signal of either H3K4me3 (K4me3) in **A.**, or H3K27me3 (K27me3) in **B.**, in *MYL6*, *HOXC* or *LINC-PINT* alleles from the same cell (one locus from each cell containing 2-4 loci is randomly assigned as allele A, and another one as allele B). Black lines represent the threshold "on" level for each histone mark fluorescence signal. The correlation coefficient (*r*) for each set is shown in the top-right corner of each plot.

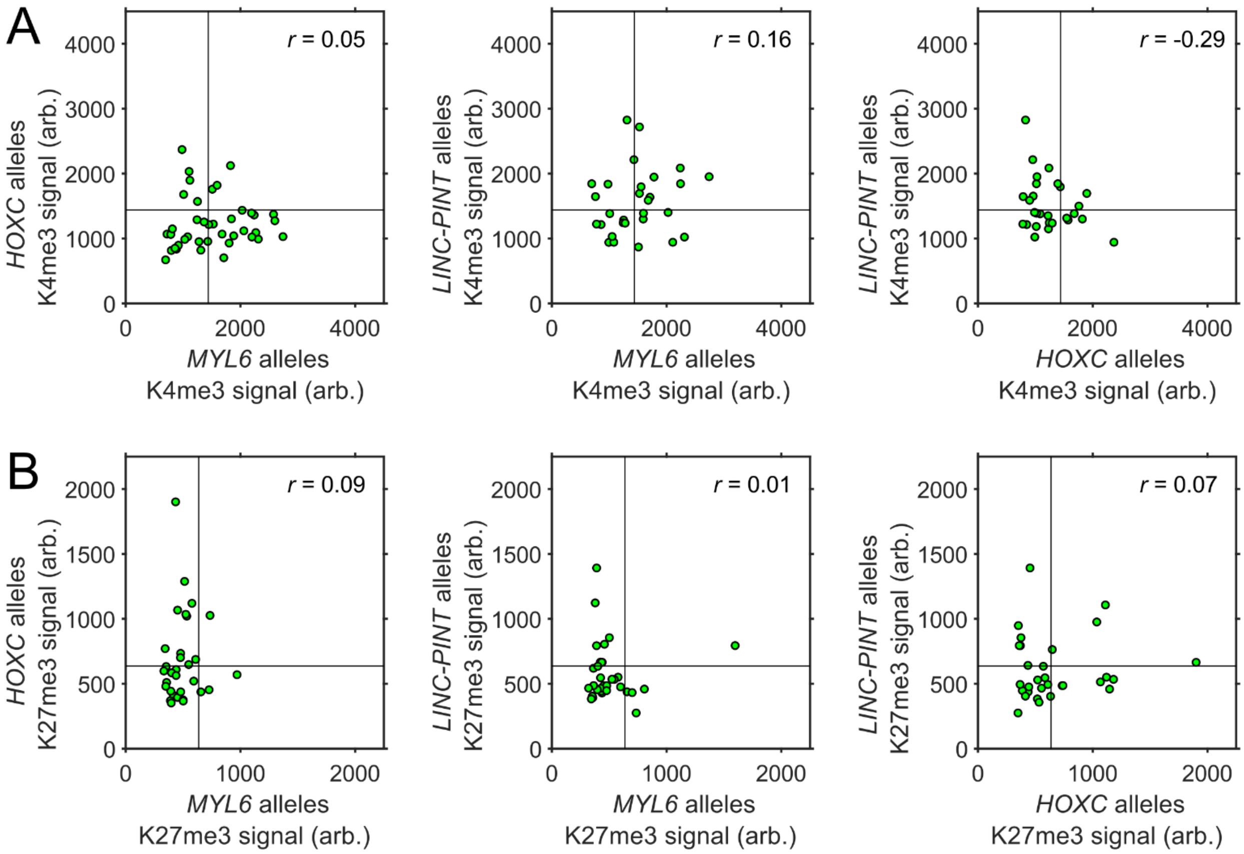

Supplementary figure 10. SCEPTRE compares H3K4me3 or H3K27me3 signals between alleles from different genes in the same cell. (A-B) Comparison of the fluorescence signal (arb. = arbitrary units) of either H3K4me3 (K4me3) in **A.,** or H3K27me3 (K27me3) in **B.**, between randomly selected alleles of *MYL6*, *HOXC* and/or *LINC-PINT* within the same cell. Black lines represent the threshold “on” level for each histone mark fluorescence signal. The correlation coefficient (*r*) for each set is shown in the top-right corner of each plot.

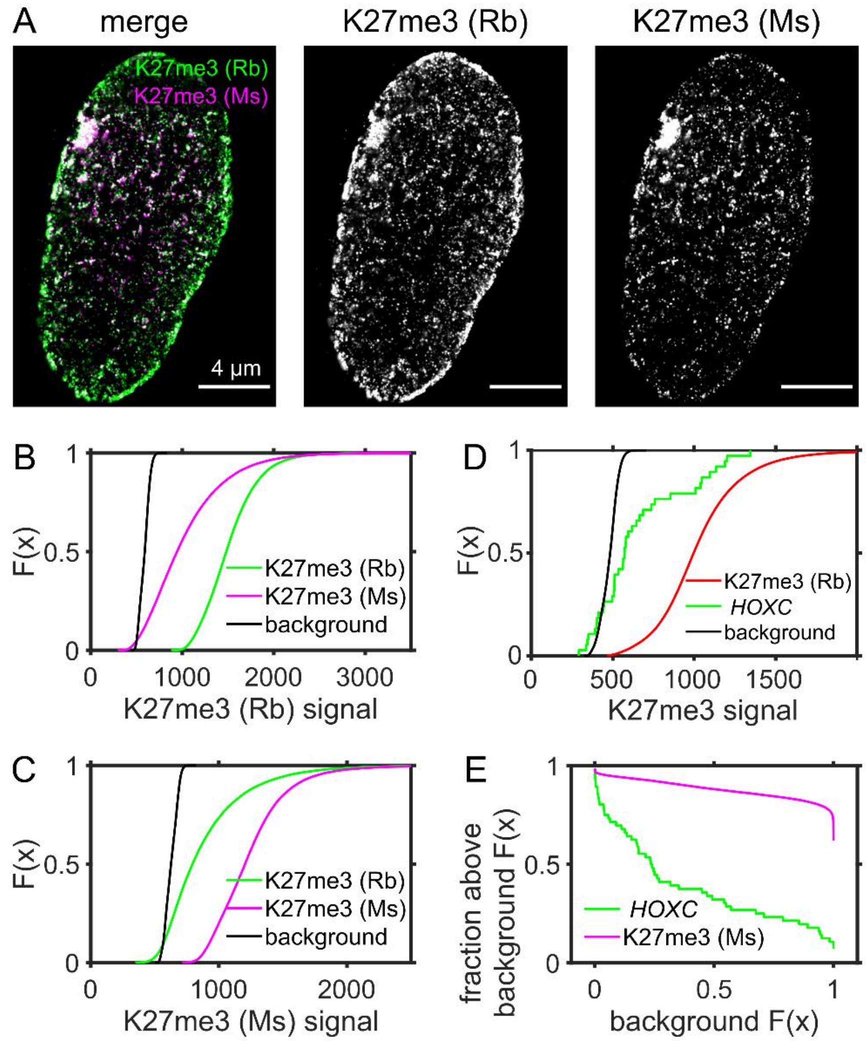

Supplementary figure 11. Dual labeling of H3K27me3 marks with two different antibodies provides information on the detection efficiency of each antibody. (A) An expanded RPE1 cell with H3K27me3 (K27me3) immunolabeled simultaneously by two different antibodies: Rb×H3K27me3 (Rb, green) and Ms×H3K27me3 (Ms, magenta). **(B-C)** Empirical cumulative distribution functions (F(x)) for the fluorescence signal of Rb×H3K27me3 in **B.**, or Ms×H3K27me3 in **C.**, for Rb×H3K27me3 clusters (green), Ms×H3K27me3 clusters (magenta), or background (black) from the same group of cells as the one in **A.** **(D)** Empirical cumulative distribution functions (F(x)) for the fluorescence signal of Rb×K27me3 in *HOXC* clusters (green), Rb×K27me3 clusters (red), or background (black) from the RPE expanded cells in **figure 4C.** **(E)** The fraction of either *HOXC* clusters (green) from **figure 4C,** or the Ms×H3K27me3 clusters (magenta) from the same group of cells as the one in **A.**, that are above the Rb×H3K27me3 fluorescence signal of background F(x).

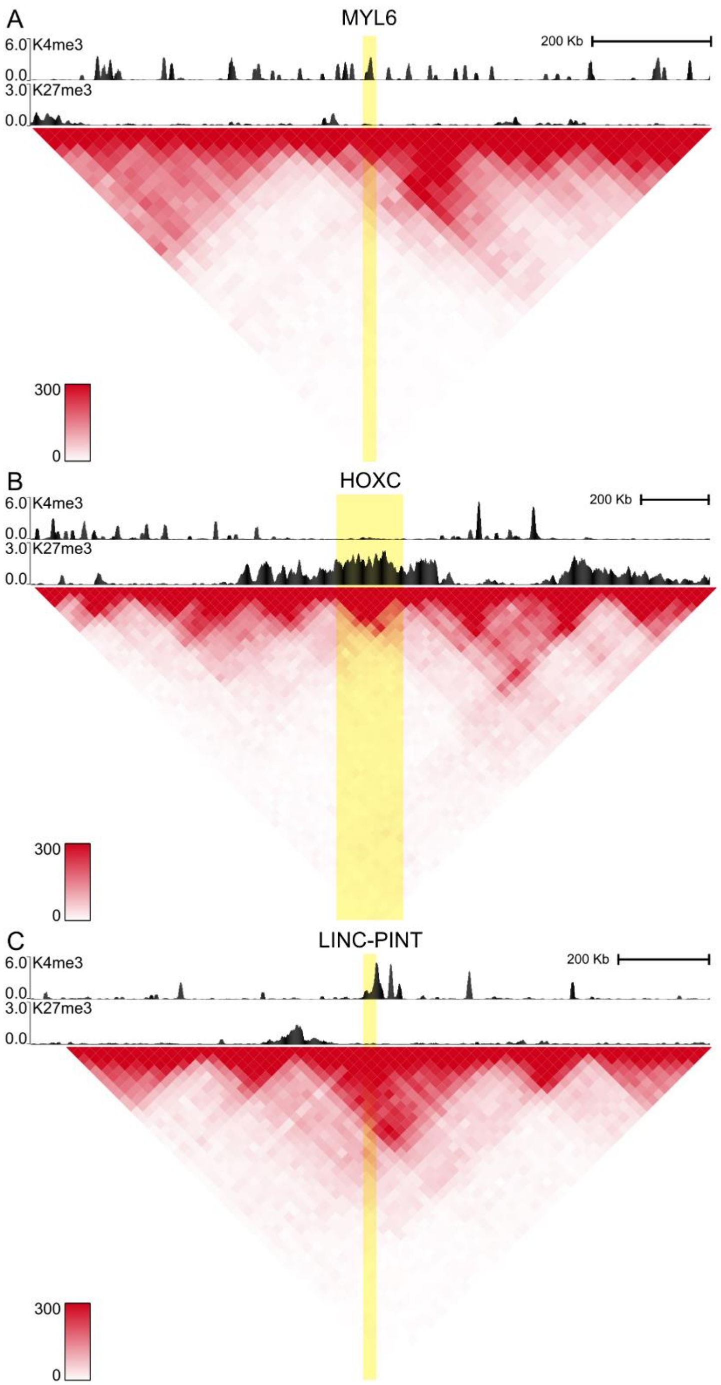

Supplementary Figure 12. Analysis of Hi-C for targeted genomic regions in RPE 1 cells. Hi-C data, previously published,(1) along with H3K4me3 (K4me3) and H3K27me3 (K27me3) CUT&RUN normalized counts for *MYL6* **(A)**, *HOXC* **(B)** and *LINC-PINT* **(C)** targeted regions (highlighted). Heat map score between 0 - 300 reads in 25 kb bins.

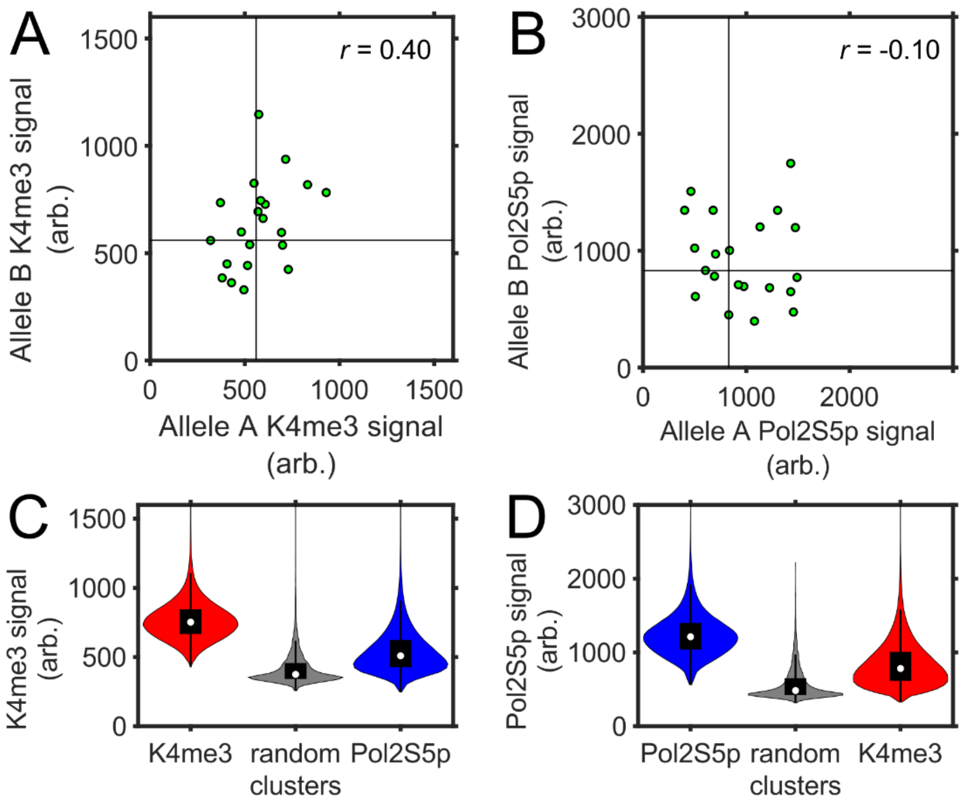

Supplementary figure 13. SCEPTRE compares H3K4me3 and paused RNA polymerase II signals between different *GAPDH* alleles in the same cell, or between immunolabeled cluster distributions. (A-B) Fluorescence signal (arb. = arbitrary units) of either H3K4me3 (K4me3) in **A.**, or paused RNA polymerase II (Pol2S5p) in **B.**, in *GAPDH* alleles within the same cell from the data set in **figure 5** (one locus from each cell containing 2-4 loci is randomly assigned as allele A, and a second locus as allele B). Black lines represent the threshold "on" level for each fluorescence signal. The correlation coefficient (*r*) is shown on the top-right corner of each plot. **(C-D)** Fluorescence signal (arb. = arbitrary units) for either H3K4me3 in **C.**, or paused RNA polymerase II in **D.**, for each distribution of H3K4me3 (red), paused RNA polymerase II (blue) and randomly selected regions (random, gray) clusters within the cells in **figure 5**.

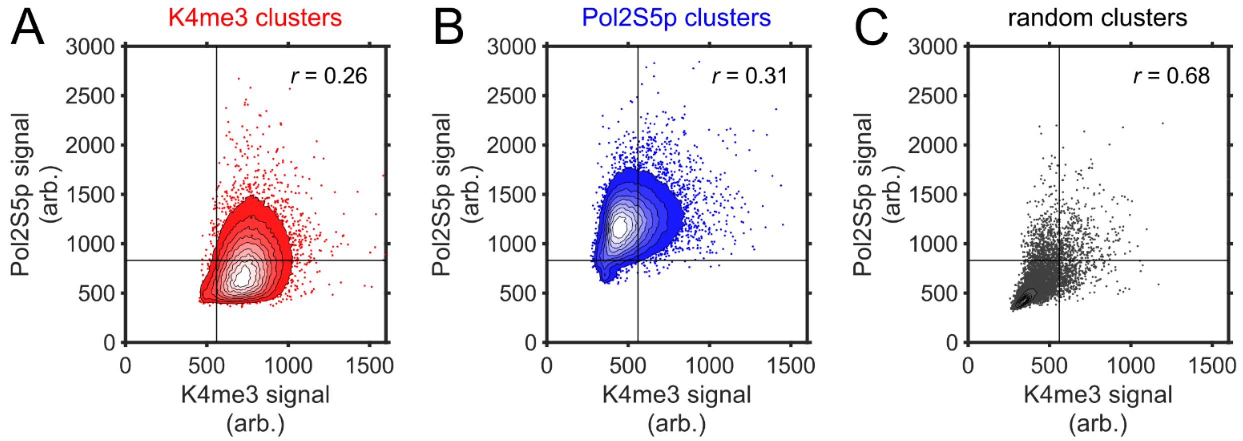

Supplementary figure 14. SCEPTRE compares H3K4me3 and paused RNA polymerase II signals within segmented immunostained and random clusters. (A-C) Contours for the fluorescence signal (arb. = arbitrary units) frequency of H3K4me3 (K4me3) and paused RNA polymerase II (Pol2S5p) in the cluster sets of H3K4me3 (red) in **A.**, paused RNA polymerase II (blue) in **B.**, and randomly selected regions (random, gray) in **C.** Straight black lines represent the threshold “on” level for each fluorescence signal. Contours have uniformly spaced steps ranging from 0.1 to 0.9 frequency and represent all clusters obtained for cells in **figure 5**. The remaining scatter in **A.** and **B.** is a 100-fold downsample of the original data by random selection for plot representation purposes. Correlation coefficients (*r*) for each data set, which are calculated before downsampling, are shown in the top-right corner of each plot.

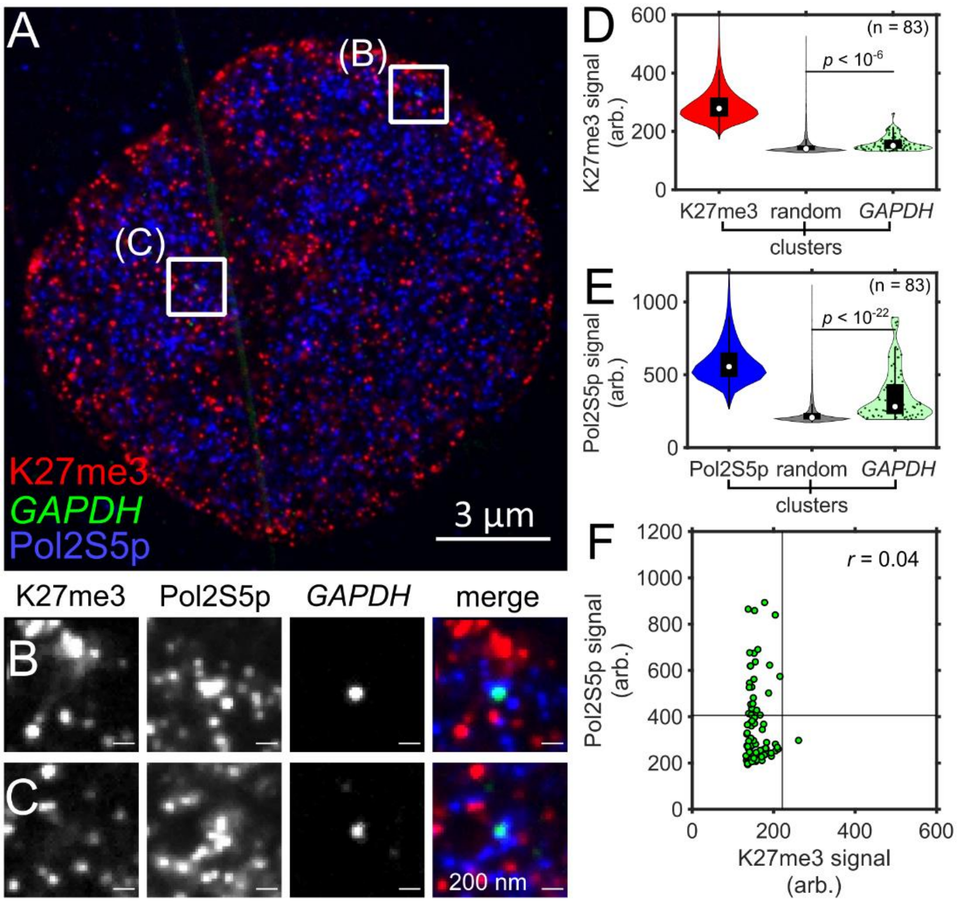

Supplementary figure 15. SCEPTRE distinguishes between H3K27me3 and paused RNA polymerase II signals at a single genomic region. (A) An expanded RPE1 cell with immunolabeled H3K27me3 (K27me3, red) and paused RNA polymerase II (Pol2S5p, blue), and FISH-labeled *GAPDH* (green). **(B-C)** Zoomed in views of the approximate center plane of an image stack for each *GAPDH* allele in the cell seen in **A. (D)** Distributions of H3K27me3 fluorescence signal (arb. = arbitrary units) within H3K27me3, randomly selected regions (random) and *GAPDH* clusters. **(E)** Distribution of paused RNA polymerase II fluorescence signal within paused RNA polymerase II, randomly selected regions and *GAPDH* clusters. **(F)** H3K27me3 and paused RNA polymerase II fluorescence signals within *GAPDH* clusters (green). Black lines represent the threshold “on” level for each fluorescence signal. Cluster numbers for **D.** and **E.** are K27me3 = 174072, Pol2S5p = 213724, random = 6099, *GAPDH* = 83. Significance determined by a right-tailed Wilcoxon rank-sum test of fluorescence signals in *GAPDH* against random cluster distributions. All scale bars are in pre-expansion units.

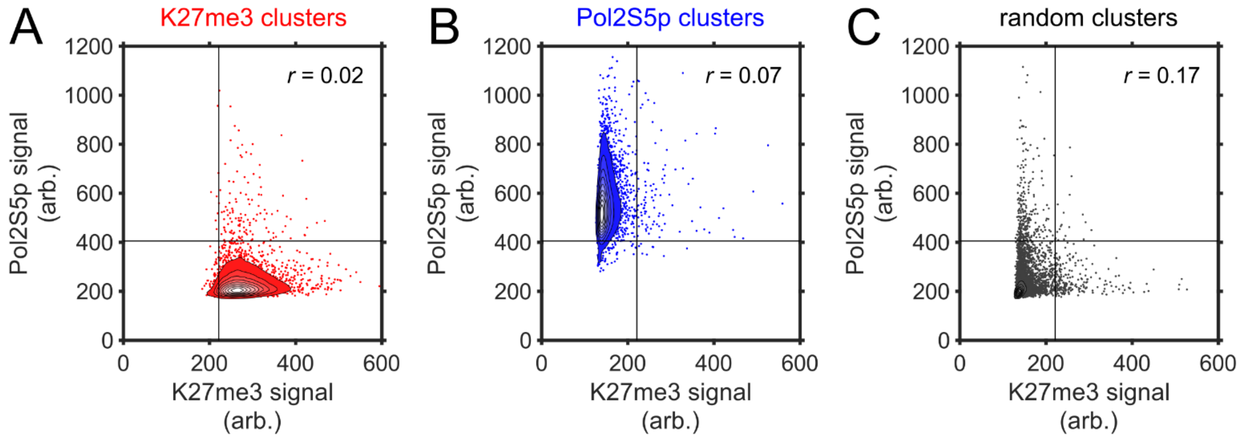

Supplementary figure 16. SCEPTRE compares H3K27me3 and paused RNA polymerase II signals within segmented immunostained and random clusters. (A-C) Contours for the fluorescence signal (arb. = arbitrary units) frequency of H3K27me3 (K27me3) and paused RNA polymerase II (Pol2S5p) in the cluster sets of H3K27me3 (red) in **A.**, paused RNA polymerase II (blue) in **B.**, and randomly selected regions (random, gray) in **C.** Straight black lines represent the threshold “on” level for each fluorescence signal. Contours have uniformly spaced steps ranging from 0.1 to 0.9 frequency and represent all clusters obtained for cells in **supplementary figure 12**. The remaining scatter in **A.** and **B.** is a 100-fold downsample of the original data by random selection for plot representation purposes. Correlation coefficients (*r*) for each data set, which are calculated before downsampling, are shown in the top-right corner of each plot.

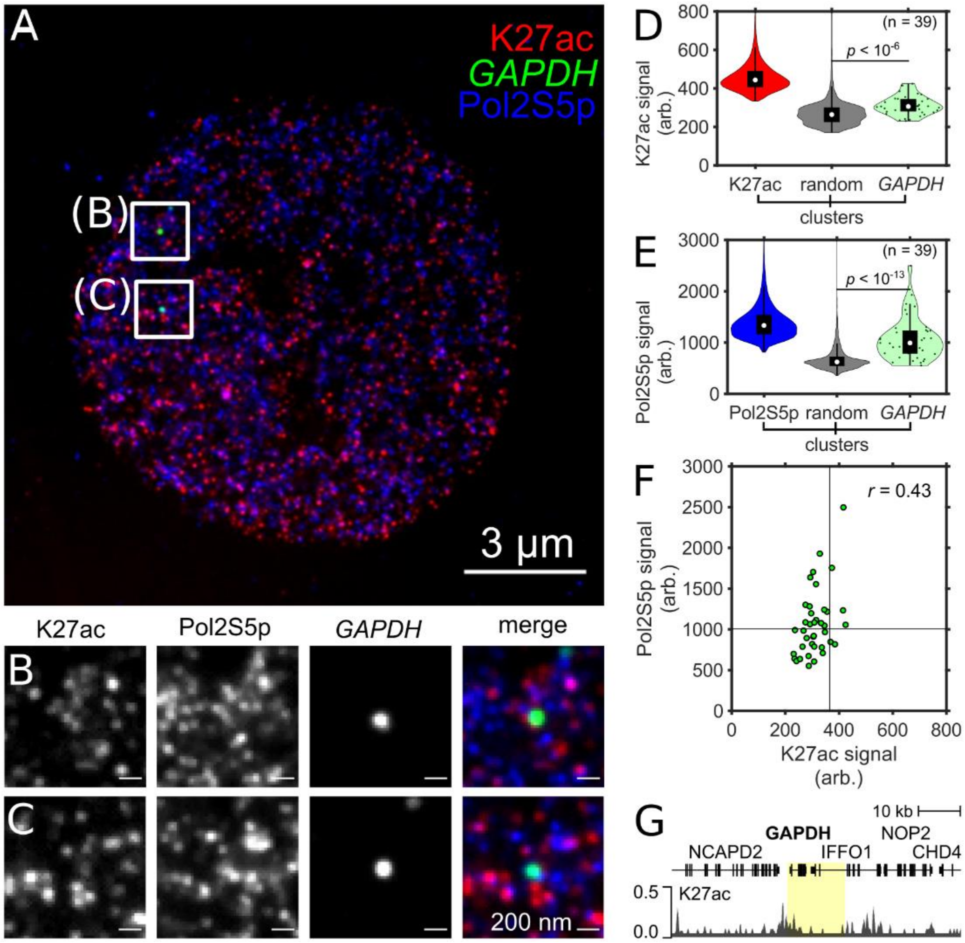

Supplementary figure 17. SCEPTRE compares H3K27ac and paused RNA polymerase II signals at a single genomic region. (A) An expanded RPE1 cell with immunolabeled H3K27ac (K27ac, red) and paused RNA polymerase II (Pol2S5p, blue), and FISH-labeled *GAPDH* (green). **(B-C)** Zoomed in views of the approximate center plane of an image stack for each *GAPDH* allele in the cell seen in **A. (D)** Distributions of H3K27ac fluorescence signal (arb. = arbitrary units) within H3K27ac, randomly selected regions (random) and *GAPDH* clusters. **(E)** Distribution of paused RNA polymerase II fluorescence signal within paused RNA polymerase II, randomly selected regions and *GAPDH* clusters. **(F)** H3K27ac and paused RNA polymerase II fluorescence signals within *GAPDH* clusters (green). Black lines represent the threshold "on" level for each fluorescence signal. (**G**) CUT&RUN normalized counts for H3K27ac marks in RPE1 cells for the FISH targeted *GAPDH* region (highlighted). Cluster numbers for **D.** and **E.** are K27ac = 82644, Pol2S5p = 153482, random = 3815, *GAPDH* = 39. Significance determined by a right-tailed Wilcoxon rank-sum test of fluorescence signals in *GAPDH* against random cluster distributions. All scale bars are in pre-expansion units.

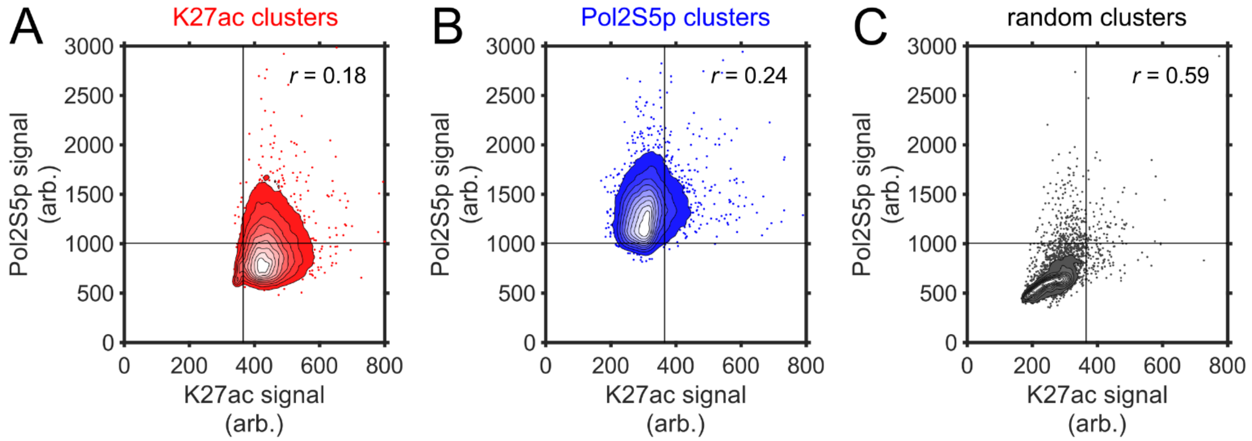

Supplementary figure 18. SCEPTRE compares H3K27ac and paused RNA polymerase II signals within segmented immunostained and random clusters. (A-C) Contours for the fluorescence signal (arb. = arbitrary units) frequency of H3K27ac (K27ac) and paused RNA polymerase II (Pol2S5p) in the cluster sets of H3K27ac (red) in **A.**, paused RNA polymerase II (blue) in **B.**, and randomly selected regions (random, gray) in **C.** Straight black lines represent the threshold "on" level for each fluorescence signal. Contours have uniformly spaced steps ranging from 0.1 to 0.9 frequency and represent all clusters obtained for cells in **supplementary figure 14**. The remaining scatter in **A.** and **B.** is a 100-fold downsample of the original data by random selection for plot representation purposes. Correlation coefficients (*r*) for each data set, which are calculated before downsampling, are shown in the top-right corner of each plot.

Supplementary tables:

Supplementary table S1. Summary of sample preparation and imaging conditions.

**Figure**	**Fixation**	**Primary ab(s).**	**Secondary ab(s).**	**Post-fixation**	**DNA FISH conditions**	**other stain:**	**Imaging**
**2**	10% PFA<br>10 min, RT	Hu × cen.<br>(5 µg/mL)	D × Hu, AT488<br>(~13 dp, 2 µg/mL)	none	Protocol: 2-step*<br>200 nM alphasat probe<br>200 nM alphasat adapter<br>600 nM AF647 reporter A<br>Denaturation: 95 °C,<br>15 min<br>Hybridization: 37 °C,<br>30 min	Hoechst<br>(1 µg/mL)<br>10 min	Microscope: LSC<br>Image thickness:<br>4.3 µm<br>buffer: 0.2× SSC<br>filter: 1
**3**	EtOH:MeOH<br>6 min, -20 °C	Rb × H3K4me3<br>(2 µg/mL)<br>Ms × H3K27me3<br>(2 µg/mL)	D × Rb, AF568<br>(~3.5 dp, 2 µg/mL)<br>D × Ms, AF488<br>(~2.5 dp, 3 µg/mL)	4% PFA<br>10 min,<br>RT	Protocol: Single-step**<br>200nM GAPDH set<br>250nM AT647N reporter B<br>Denaturation: 90 °C,<br>2.5 min<br>Hybridization: 42 °C, ON	none	Microscope: SDC<br>Image thickness:<br>211 nm<br>buffer: water<br>filter: none
**4A**	4% PFA<br>10 min, RT	Rb × H3K4me3<br>(2 µg/mL)	D × Rb, AF488<br>(~2.5 dp, 3 µg/mL)	none	Protocol: Single-step**<br>~4 µM oligo pool***<br>240 nM MYL6 Adapter<br>250 nM AF750 reporter C<br>250 nM LINC-PINT adapter<br>250 nM AF647 reporter D<br>1.25 µM HOXC adapter<br>1.25 µM AT565 reporter E<br>Denaturation: 90 °C,<br>2.5 min<br>Hybridization: 42 °C, ON	none	Microscope: SDC<br>Image thickness:<br>261 nm<br>buffer: ALOX****<br>filter: none
**4C**	Same as<br>Fig. 4A	Rb × H3K27me3<br>(2 µg/mL)	Same as Fig. 4A	none	same as fig. 4A	none	Same as Fig. 4A
**5**	Same as<br>Fig. 3	Rb × H3K4me3<br>(2 µg/mL)<br>Ms × Pol2S5p<br>(2 µg/mL)	D × Rb, AF568<br>(~2.7 dp, 3 µg/mL)<br>D × Ms, AF488<br>(~2.5 dp, 3 µg/mL)	Same as<br>Fig. 3	Protocol: Single-step**<br>100 nM GAPDH set<br>100 nM AT647N reporter B<br>Denaturation: 90 °C,<br>2.5 min<br>Hybridization: 42 °C, ON	none	Same as Fig. 3
**S1**	Same as<br>Fig. 3	Same as Fig. 3	D × Rb, AF568<br>(~3.3 dp, 2 µg/mL)<br>D × Ms, AF488<br>(~2.5 dp, 2 µg/mL)	none	none	none	Microscope:<br>Widefield<br>Image thickness: 1 µm<br><br>buffer: 0.2× SSC<br>filter: 1
**S3**	Same as<br>Fig. 2	Same as Fig. 2	Same as Fig. 2	none	none	Same as<br>Fig. 2	Microscope: LSC<br>Image thickness:<br>5.3 µm<br>buffer: water<br>filter: 1

**S4A**	Same as Fig. 4A	Same as Fig. 4A	Same as Fig. 4A	none	Protocol: Single-step**<br>200 nM GAPDH set<br>300 nM AT565 reporter B<br>Denaturation: 92.5 °C, 10 min<br>Hybridization: 37 °C, ON	none	Microscope: LSC<br>Image thickness: 225 nm<br>buffer: water<br>filter: none
**S4B**	Same as Fig. 4A	Same as Fig. 4B	Same as Fig. 4A	none	Same as Sup. Fig. 3A	none	Microscope: SDC<br>Image thickness: 206 nm<br>buffer: water<br>filter: none
**S5**	Same as Fig. 3	Ms × H3K4me3 (2 µg/mL)<br>Rb × H3K27me3 (2 µg/mL)	Same as Fig. 3	Same as Fig. 3	Same as Fig. 3	none	Same as Fig. 3
**S7**	Same as Fig. 3	Rb × H3K4me3 (2 µg/mL)<br>Ms × H3K4me3 (2 µg/mL)	Same as Fig. S1	Same as Fig. 3	None	None	Same as Fig. S4B
**S11**	Same as Fig. 3	Rb × H3K27me3 (2 µg/mL)<br>Ms × H3K27me3 (2 µg/mL)	Same as Fig. S1	Same as Fig. 3	None	None	Same as Fig. S4B
**S13**	Same as Fig. 3	Rb × H3K27me3 (2 µg/mL)<br>Ms × Pol2S5p (2 µg/mL)	Same as Fig. 5	Same as Fig. 3	Protocol: Single-step**<br>100 nM GAPDH set<br>120 nM AT647N reporter B<br>Denaturation: 90 °C, 2.5 min<br>Hybridization: 42 °C, ON	none	Same as Fig. 3
**S15**	Same as Fig. 3	Rb × H3K27ac (2 µg/mL)<br>Ms × Pol2S5p (2 µg/mL)	Same as Fig. 5	Same as Fig. 3	Same as Sup. Fig. 12	Same as Fig. 2	Same as Fig. 3

* 2-step protocol: alpha-satellite probe is hybridized first after denaturation, and after the sample is washed, adapter and reporter probes are hybridized in a second step.

** single-step protocol: All probes are hybridized together in one step.

*** 4 µM oligo pool is assumed to contain ~180 nM *MYL6* probe set, ~244 nM *LINC-PINT* probe set and ~1.2 µM *HOXC* probe set.

**** ALOX buffer contains: 10 mM Tris buffer (pH 8) with 1 mM Methyl viologen, 1 mM Ascorbic acid, 2% (v/v) MeOH, ~30 units/mL alcohol oxidase and 0.2% (w/v) catalase.

<u>Additional notes:</u> Thickness in the imaging column refers to the thickness of the displayed data in terms of the pixel sizes set in pre-expansion units. Filter refers to the number of pixels used in applying a 3D median filter to the image data set before being displayed in the figure.

<u>Acronyms</u>: PFA=Paraformaldehyde; RT=room temperature (~22 °C); ab=antibody; Hu=Human; Rb=Rabbit; Ms=Mouse; D=Donkey; dp=dyes per protein; ON=Overnight (~18 hours); AF=Alexa Fluor; AT=ATTO-TEC; alphasat=alpha-satellite; LSC=Laser Scanning Confocal; SDC = Spinning Disk Confocal.

Supplementary table S2. Summary of image processing and analysis conditions.

**Figure**	**nuclear mask channel**	**gaussian smooth (SD)**	**contrast adjustment threshold**	**Binarization method**	**FISH size filter (voxels)**
**2**	Hoechst	2	nuclear: 1<br>α-centromere: 2<br>anti-centromere: 5	Otsu	α-cen. size: 20 – 10000
**3**	H3K27me3	1	nuclear: 3<br>GAPDH: 10<br>H3K4me3: 3<br>H3K27me3: 2	Laplace	GAPDH: ≥20
**4A**	H3K4me3	1	nuclear: 2.5<br>MYL6: 10<br>HOXC: 10<br>LINC-PINT: 10<br>H3K4me3: 2.5	Laplace	MYL6: ≥20<br>HOXC: ≥20<br>LINC-PINT: ≥20
**4C**	H3K27me3	1	nuclear: 3<br>MYL6: 10<br>HOXC: 10<br>LINC-PINT: 10<br>H3K27me3: 3	Laplace	MYL6: ≥20<br>HOXC: >50<br>LINC-PINT: >50
**5**	Pol2S5p	1	nuclear: 3<br>GAPDH: 15<br>H3K4me3: 5<br>Pol2S5p: 5	Laplace	GAPDH: >50
**S4A**	H3K4me3	1	nuclear: 4<br>GAPDH: 15<br>H3K4me3: 3	Laplace	GAPDH: >80
**S4B**	H3K27me3	1	nuclear: 3:<br>GAPDH: 15<br>H3K27me3: 3	Laplace	GAPDH: >80
**S5**	H3K27me3	1	Nuclear: 3<br>GAPDH: 10<br>H3K4me3: 3<br>H3K27me3: 3	Laplace	GAPDH: >50
**S7**	H3K4me3 (Rb)	1	Nuclear: 2<br>H3K4me3 (Rb): 3<br>H3K4me3 (Ms): 3	Laplace	none
**S11**	H3K27me3 (Rb)	1	Nuclear: 2<br>H3K27me3 (Rb): 3<br>H3K27me3 (Ms): 3	Laplace	None
**S14**	H3K27me3	1	Nuclear: 3<br>GAPDH: 15	Laplace	GAPDH: >75

			H3K27me3: 9<br>Pol2S5p: 9		
**S16**	Hoechst	1	Nuclear: 1<br>GAPDH: 10<br>H3K27ac: 2<br>Pol2S5p: 4	Laplace	GAPDH: ≥20

Acronym: SD=standard deviation

Additional notes: Contrast adjustment threshold represents the number of third quartiles above the median of an image stack histogram used to establish the threshold for clipping during contrast adjustment (see Materials and Methods for more details).

Supplementary table 3. Transcription levels for genes found within DNA FISH-labeled genes profiled by SCEPTRE.

DNA FISH-labeled region	gene within labeled region	Transcription level (FPKM)*
GAPDH	GAPDH	1618.65
	IFFO1	3.00
MYL6	MYL6	928.92
	MYL6B	19.73
	SMARCC2	10.09
HOXC	HOXC4	0.00
	HOXC5	0.01
	HOXC6	0.00
	HOXC8	0.00
	HOXC9	0.00
	HOXC10	0.00
	HOXC11	0.00
	HOXC12	0.00
	HOXC13	0.00
	HOTAIR	0.00
	HOXC-AS1	0.00
	HOXC-AS2	0.00
	HOXC-AS3	0.00
LINC-PINT	LINC-PINT	2.59

*Results obtained in previous RNA-seq study performed on RPE1 cells.(2)

Supplementary references:

1. Darrow,E.M., Huntley,M.H., Dudchenko,O., Stamenova,E.K., Durand,N.C., Sun,Z., Huang,S.-C., Sanborn,A.L., Machol,I., Shamim,M., *et al.* (2016) Deletion of DXZ4 on the human inactive X chromosome alters higher-order genome architecture. *PNAS*, **113**, E4504–E4512.

2. Harenza,J.L., Diamond,M.A., Adams,R.N., Song,M.M., Davidson,H.L., Hart,L.S., Dent,M.H., Fortina,P., Reynolds,C.P. and Maris,J.M. (2017) Transcriptomic profiling of 39 commonly-used neuroblastoma cell lines. *Scientific Data*, **4**, 170033.
